# The internal dose makes the poison: higher internalization of polystyrene particles induce increased perturbation of macrophages

**DOI:** 10.3389/fimmu.2023.1092743

**Published:** 2023-05-12

**Authors:** Véronique Collin-Faure, Marianne Vitipon, Anaëlle Torres, Ornella Tanyeres, Bastien Dalzon, Thierry Rabilloud

**Affiliations:** Chemistry and Biology of Metals, Univ. Grenoble Alpes, CNRS UMR5249, CEA, IRIG-LCBM, Grenoble, France

**Keywords:** microplastics, polystyrene, macrophages, oxidative stress, mitochondria, integrins

## Abstract

Plastics are emerging pollutants of great concern. Macroplastics released in the environment degrade into microplastics and nanoplastics. Because of their small size, these micro and nano plastic particles can enter the food chain and contaminate humans with still unknown biological effects. Plastics being particulate pollutants, they are handled in the human body by scavenger cells such as macrophages, which are important players in the innate immune system. Using polystyrene as a model of micro and nanoplastics, with size ranging from under 100 nm to 6 microns, we have showed that although non-toxic, polystyrene nano and microbeads alter the normal functioning of macrophages in a size and dose-dependent manner. Alterations in the oxidative stress, lysosomal and mitochondrial functions were detected, as well as changes in the expression of various surface markers involved in the immune response such as CD11a/b, CD18, CD86, PD-L1, or CD204. For each beads size tested, the alterations were more pronounced for the cell subpopulation that had internalized the highest number of beads. Across beads sizes, the alterations were more pronounced for beads in the supra-micron range than for beads in the sub-micron range. Overall, this means that internalization of high doses of polystyrene favors the emergence of subpopulations of macrophages with an altered phenotype, which may not only be less efficient in their functions but also alter the fine balance of the innate immune system.

## 1 Introduction

The technical properties of plastics (light weight, mechanical resistance, resistance to water) has promoted their wide use in a myriad of products, resulting in annual production figures amounting in a fraction of a gigaton (1). A huge proportion of these plastics (estimated to 500Mt/year (2)) ends up in the environment, and progressively enter the food chain (3) as the plastics degrade into micro and nanoplastics. This eco-contamination of food products e.g. seafood, superposes to direct abrasion of plastic food containers and packaging to contaminate humans. The current plastic ingestion is estimated to be up to 5g of plastic particles per week (4). Indeed, microplastics have been observed in human feces (5), but also in human tissues such as lungs (6), liver (7) or placenta (8). This poses in turn the question of how the human body defends itself against plastics, and what physiological reactions these plastic particles induce.

In this context the innate immune system *sensu lato* (i.e. including the epithelial barriers) plays a major role in protecting the body against plastic particles. As ingestion is the first contamination route that has been identified, many studies have focused on intestinal effects of plastic, both using *in vivo* (9) and *in vitro* models (e.g. in (10–12)). In this respect, it has been shown that a fraction of the plastic particles coming into contact with the intestinal barrier are able to translocate through it, reaching deeper organs such as liver, kidney (9) blood (13) or placenta (8).

Once particles have crossed the first line of defense of the body, such as epithelial barrier or the upper airways filters, macrophages represent the second line of defense. They capture almost any type of particular material, up to 10µm in diameter (14, 15), to minimize the contamination of the rest of the body to particulate material, helping at the homeostasis of the whole body (16). Indeed, macrophages are found at many critical locations such as the liver (Kupffer cells), lymphoid organs (spleen and lymph nodes), alveolae, gastrointestinal tract, dermis and brain (microglia).

Macrophages respond to the particles that they internalize, and alter their physiology according to the nature of the particles. The most canonical examples are the responses to bacteria on the one hand and apoptotic bodies on the other hand, which induce what is called macrophage polarization in different final physiological states, respectively called M1 (for bacteria) and M2 (for apoptotic bodies) (see (17) for review). These states are characterized by various markers (18, 19), including (but not limited to) surface markers such as CD38 for the M1 state (18) and CD204 for the M2 state (20, 21). This opens the question of the reaction that plastics may trigger on macrophages. Previous studies have shown that polystyrene, for example, induces a weak pro-inflammatory phenotype (22, 23), and alters the phenotype of pre-polarized macrophages (24).

These effects have been shown to be plastic concentration-dependent (22, 23). More recently, results on the effects of polystyrene beads of different sizes on macrophages have suggested that some effects may be dependent on the amount of plastic internalized by the cells (25). We have therefore decided to investigate this concept further, and tried to determine if the cell population heterogeneity leads to different reactions to the exposure to micro or nanoplastics. The underlying question is to determine if, upon exposure to micro or nanoplastics, a subpopulation of macrophages acquires new characteristics that may, on the long term, alter the response of the innate immune system.

This question is a general one for many, if not all, biopersistent particles, which are eliminated very slowly from the body. After their capture by macrophages, they are released upon the death of the macrophages and recaptured by new macrophages, ensuring their persistence in the body. This has been shown to occur with Kupffer cells (26), but also with dermal macrophages, the latter mechanism ensuring the persistence of tattoos over the lifetime (27). Consequently, if some particles, in our case plastic particles, skew the phenotype of the macrophages that have internalized them, this skewing may persist over time in the body and thus alter the homeostasis of the innate immune system. Such an effect has been demonstrated *in vivo* with titanium dioxide (28, 29), and recent results obtained *in vitro* suggest that this may happen with other mineral persistent particles (30).

We therefore decided to investigate how macrophages respond to the ingestion of plastic particles. As the plastic source, we decided to use polystyrene. Polystyrene is used for manufacturing items used for food of beverage consumption, and is known to represent a fair proportion of the amount of plastics found in the environment (31). It is also known to degrade into nanoplastics (32), and even seems to be more prevalent in the nanoplastic fraction than in the microplastic one (33). It is commercially available as beads of different sizes, which is important as nano and microplastics coming from large plastics erosion of fragmentation cover a wide range of sizes too (33). We decided to cover sizes from 70nm in diameter to up to 7µm, as particles of a few micrometers in size have been shown to be able to cross the intestinal barrier, although with a low efficiency (10, 34–36).

## 2 Material and methods

### 2.1 Plastic particles

Fluorescent spherical plain polystyrene particles (d=1.05) were used. Skyblue-dyed polystyrene particles were purchased from Spherotech with nominal diameters of 40-90 nm (catalog number #FP-00570-2), 0.7-0.9µm (catalog number #FP-00870-2), 1.7-2.2 µm (catalog number #FP-02070-2), 2.5-4.5 µm (catalog number #FP-04070-2), and 6-8 µm (catalog number #FP-6068-2). These particles were excited at 561 nm and their fluorescence read at 695 nm. The chemical composition of the beads was verified by FTIR spectroscopy.

For size characterization, the Spherotech bead suspensions were diluted in water to 100µg/ml. For the observation and measurement by optical microscopy (micrometric particles, 0.7µm and higher) we used a graduated slide and 20µl of the diluted suspension was deposited on it. A representative picture was taken for each of the suspensions, then the size of the beads were measured using the ImageJ software compared with the known distance between the graduations of the slide. The size of the nanometric particles (40-90 nm) (by dynamic light scattering) and the zeta potential were measured with a Malvern zeta sizer (parameters: Smoluchowski F(Ka) model, polystyrene latex material, 25°C, in water). For each suspension, 3 measurements were performed to avoid bias due to sedimentation. The size of the nanometric beads was measured with the DLS program of this instrument, and 3 measurements were performed.

For characterization of the behavior of the particles in culture medium over time, the Spherotech bead suspensions were diluted in complete culture medium (DMEM+ 10% serum, filtered through a 0.22µm pore size filter) at 100µg/ml (0.7-0.9 µm beads) or 20µg/ml (other beads sizes) and incubated at 37°C for 24 hours. The beads were then characterized by the same optical methods as described above.

### 2.2 Cell culture and exposure

The J774A.1 cell line (mouse macrophages) was purchased from European cell culture collection (Salisbury, UK), and used at passage numbers 5-15 post reception from the repository. Cells were routinely propagated in DMEM supplemented with 10% fetal bovine serum (FBS) in non-adherent flasks (Cellstar flasks for suspension culture, Greiner Bio One, Les Ulis, France). For routine culture, the cells were seeded at 200,000 cells/ml and passaged two days later, with a cell density ranging from 800,000 to 1,000,000 cells/ml. For exposure to plastic particles and to limit the effects of cell growth, cells were seeded at 500,000 cells/ml, let settle and recover for 24 hours, and then exposed to the particles at 20µg/ml for 24 hours before harvesting for the experiments. For some experiments, colloidal silica particles (50 nm from Micromod, #43-00-501, used at a 50 µg/ml concentration) were used as a model of particles inducing a pro-inflammatory response (37).

For assessing the persistence of the particles in the cells, they were seeded in adherent 12-well plates at 300,000 cells/ml in DMEM supplemented with 1% horse serum, in order to limit their proliferation (38). After adaptation to the medium for 72 hours, they were exposed to the plastic particles for 24 hours at a concentration of 20 µg/ml. The particle-containing medium was removed, half of the wells were harvested and analyzed by flow cytometry to determine particle uptake. The remaining cells were cultivated in DMEM supplemented with 1% horse serum for an additional 9 days without splitting, and with a culture medium change every 2 days. The cells were then harvested and analyzed by flow cytometry. In order to normalize for the cell number, a cell numeration was performed on the harvested cells in each well.

### 2.3 Confocal microscopy

For confocal microscopy experiments, the cells were seeded on glass coverslips and let adhere for 24 hours prior to the double exposure. At the end of the exposure period, the cells were washed twice for 5 min in PBS, fixed in 4% paraformaldehyde for 30 min at room temperature. After two washes (5 min in PBS), they were permeabilized in Triton X100 (0.1% w/v) for 5 min at room temperature. After two more washes in PBS, Phalloidin-Atto 550 (Sigma) (200 nM) or Phalloidin-Atto 390 (500 nM) was added to the cells and let for 20 min at room temperature in the dark. Coverslips-attached cells were washed, and cell nuclei were colored via DAPI for 5 minutes at room temperature, protected from light (1µg/ml, Eurobio, Les Ulis, France). The coverslips were washed twice and placed on microscope slides (Thermo Scientific, Illkirch, France) using a Vectashield mounting medium and imaged using a Zeiss LSM 880 confocal microscope. The images were processed using the ImageJ software.

### 2.4 Assays for mitochondria and lysosomes

For the mitochondrial transmembrane potential assay, Rhodamine 123 (Rh123) was added to the cultures at a 80 nM final concentration, which were further incubated at 37°C for 30 minutes. At the end of this period, the cells were collected, washed in cold PBS containing 0.1% glucose, resuspended in PBS glucose and analyzed for the green fluorescence (excitation 488 nm emission 525nm) on a Melody flow cytometer. As a positive control, butanedione monoxime (BDM) was added at a 30mM final concentration together with the Rh123 (39). As a negative control, carbonyl cyanide 4-(trifluoromethoxy)phenylhydrazone (FCCP) was added at 5µM final concentration together with the Rh123 (39).

For the lysosomal function assay, the Lysosensor method was used. After exposure to plastic beads, the medium was removed, the cell layer was rinsed with complete culture medium and incubated with 1µM Lysosensor Green (Molecular Probes) diluted in warm (37°C) complete culture medium for 1 hour at 37°C. At the end of this period, the cells were collected, washed in cold PBS containing 0.1% glucose, resuspended in PBS glucose and analyzed for the green fluorescence (excitation 488 nm emission 540nm) on a Melody flow cytometer.

### 2.5 Assay for oxidative stress

For the oxidative stress assay, a protocol based on the oxidation of dihydrorhodamine 123 (DHR123) was used. After exposure to plastic beads, the cells were treated in PBS containing 500 ng/ml DHR123 for 20 minutes at 37°C. The cells were then harvested, washed in cold PBS containing 0.1% glucose, resuspended in PBS glucose and analyzed for the green fluorescence (same parameters as rhodamine 123) on a Melody flow cytometer. Menadione (applied on the cells for 2 hours prior to treatment with DHR123) was used as a positive control in a concentration range of 25-50µM.

### 2.6 Cell surface markers

Cells were seeded into 12-well plates at a concentration of 1,000,000 cells/ml, and exposed to the plastic particles as described above. For some experiments, LPS was then added at a concentration of 100 ng/ml 6 hours after the exposure to plastic particles. The following day, the cells were harvested and washed in PBS containing 3% FBS and 0.016% (w/v) sodium azide (PBS-fluo). The cells were then treated with the fluorochrome-conjugated antibodies at the adequate dilution for 30 minutes on ice in PBS-fluo in a final volume of 100µl. The cells were then washed with 2ml of PBS. The cell pellet was taken in 400 µl of PBS containing 1 µM Sytox blue for checking cell viability, and the suspension analyzed by flow cytometry on a Melody flow cytometer. First, live cells (Sytox blue-negative) were selected at 450 nm (excitation at 405 nm). The gated cells were then analyzed for the beads fluorescence (excitation 561nm emission 695 nm) and for the antibody fluorescence (excitation 488 emission 527 nm for FITC or A488 labelled antibodies or excitation 561 nm emission 582 nm for PE-conjugated antibodies). Isotypic control antibodies were used to compensate for non-specific binding. The following antibodies were used:

CD11a-FITC (BD-Pharmingen #553-120) dilution 1/100
CD11b-FITC (BD-Pharmingen #553-370) dilution 1/50
CD18-FITC (BD-Pharmingen #553-292) dilution 1/200
CD74-FITC (BD-Pharmingen #561-941) dilution 1/25
CD86-FITC (BD-Pharmingen #553-691) dilution 1/50
CD204-A488 (Invitrogen #53-2046-82) dilution 1/50
MARCO-FITC (R&D systems #FAB2956F) dilution 1/25
MHC1-PE (BD-Pharmingen #566-776) dilution 1/100
MHC2-FITC (Invitrogen #11-5321-82) dilution 1/25
PD-L1-A488 (BD-Pharmingen #566-864) dilution 1/100

For TLR7 (conjugated to PE, BD-Pharmingen #565-557), the cells were collected after exposure to the plastic beads, washed with PBS, and then fixed and permeabilized with the BD cytofix-cytoperm kit (#554715). The cells were incubated with the anti-TLR7 antibody diluted to 1/50 for 30 minutes on ice. The cells were then washed with the permeabilization buffer, resuspended in PBS and analyzed by flow cytometry as described above.

## 3 Results

### 3.1 Particle characterization

The results of the beads characterization are presented in **Table 1** and in **Supplementary Figure 1**. The zeta potential was negative for all the latex beads with similar values, ranging from -49 to -67mV with important standard deviation. This means that the affinity of the macrophages shall not be different whatever the size of the beads, and the possible difference of internalization throughout the conditions is likely due to the size of the latex bead. The size of the nanometric beads was determined around 72 nm with a low polydispersity index, in line with the specifications of the supplier. The FTIR spectra were consistent with previously published spectra for polystyrene (40). The images used for characterization by optical microscopy are presented in **Supplementary Figure 2**.

**Table 1:** Latex beads characterization in water, with a Malvern zeta sizer or by optical microscopy.

Spherotech SkyBlue beads in water	Nominal size range	DLS (nm)	Microscopic observation (µm)	zeta potential (mV)
	40-90 nm	71.8 ± 5.0	N/A	-48.87 ± 11.2
	0.7-0.9 µm	N/A	0.94 ± 0.17	-53.9 ± 34.8
	1.7-2.2 µm	N/A	2.02 ± 0.22	-62.83 ± 10.4
	2.5-4.5 µm	N/A	3.3 ± 0.39	-54.47 ± 13.9
	6-8 µm	N/A	6.06 ± 0.98	-66.5 ± 19.3

DLS, Dynamic light scattering; N/A, non-applicable.

Control experiments showed that the fluorophore leakage in the medium over the 24 hours incubation period was negligible (less than 1%, **Supplementary Table 1**).

In order to study the potential aggregation of the beads in the culture conditions, the sizing experiments were repeated after a 24 hours incubation in complete culture medium and the results are presented in **Table 2**. No important aggregation of the beads could be detected at any size. The small increase in the average diameter detected for some beads (e.g. in the 40-90 nm range) can be explained by the formation of a protein corona on the beads.

**Table 2:** Latex beads characterization in water, with a Malvern zeta sizer or by optical microscopy.

Spherotech SkyBlue beads in culture medium	Nominal size range	DLS (nm)	Microscopic observation (µm)
	40-90 nm	90 ± 6.63	N/A
	0.7-0.9 µm	N/A	0.87 ± 0.07
	1.7-2.2 µm	N/A	2.66 ± 0.49
	2.5-4.5 µm	N/A	3.32 ± 0.31
	6-8 µm	N/A	6.39 ± 0.86

DLS, Dynamic light scattering; N/A, non-applicable.

### 3.2 Particle internalization and persistence in macrophages

We then checked if the particles were internalized by macrophages and if they were persistent over time. To this purpose we first used flow cytometry, and measured for each well the number of cells, the proportion of cells having internalized beads (phagocytic cells) and the mean fluorescence (MFI) of the phagocytic cells. These measurements were carried out immediately after a 24 hours exposure to the plastic beads or after the same exposure followed by a 9 days recovery period. The results are presented in **Table 3**, and show that the fluorescence is almost constant when comparing cells just after exposure and cells after the nine days recovery period.

**Table 3:** Beads internalization and persistence over time.

Cell numeration D1 (in million cells)		MFI D1	%phagocytic cells D1	total fluorescence for phagocytic cells in one well D1
CTL	1.45 ± 0.14	0	4.47 ± 0.16	0
40-90 nm beads	1.30 ± 0.14	882 ± 77	99.13 ± 0.55	1137 ± 159
0.7-0.9 µm beads	1.35 ± 0.04	8246 ± 835	93.25 ± 1.64	10393 ± 983
1.7-2.2 µm beads	1.12 ± 0.28	3223 ± 63	50.43 ± 1.55	1822 ± 409
2.5-4.5 µm beads	1.01 ± 0.30	39082 ± 1055	23.54 ± 0.85	9354 ± 3123
6-8 µm beads	0.90 ± 0.15	31381 ± 1316	9.47 ± 1.26	2651 ± 409
**Cell numeration D10 (in million cells)**		**MFI D10**	**%phagocytic cells D10**	**total fluorescence phagocytic for cells in one well D10**
CTL	2.63 ± 0.20	0.0	6.20 ± 0.54	0
40-90 nm beads	2.39 ± 0.41	592 ± 28	94.16 ± 1.15	1341 ± 281
0.7-0.9 µm beads	2.59 + 0.40	5354 ± 399	85.95 ± 1.51	11964 ± 2323
1.7-2.2 µm beads	2.23 ± 0.36	2405 ± 108	37.39 ± 4.40	1999 ± 357
2.5-4.5 µm beads	2.22 ± 0.30	32133 ± 432	16.73 ± 0.83	11885 ± 1333
6-8 µm beads	2.23 ± 0.35	22607 ± 748	9.27 ± 0.90	4705 ± 1150

Indeed, there was a decrease in the MFI for the cells after the nine days recovery period, but this was compensated by the increase of the cells numbers in the wells, indicating that the decrease in MFI could be explained by signal dilution due to particles sharing during mitosis. A slight increase in signal observed when comparing the fluorescence after recovery to the one immediately after exposure, but this change was not statistically significant, except for the largest beads where the proportion of phagocytic cells is very low.

**Figure 1:**
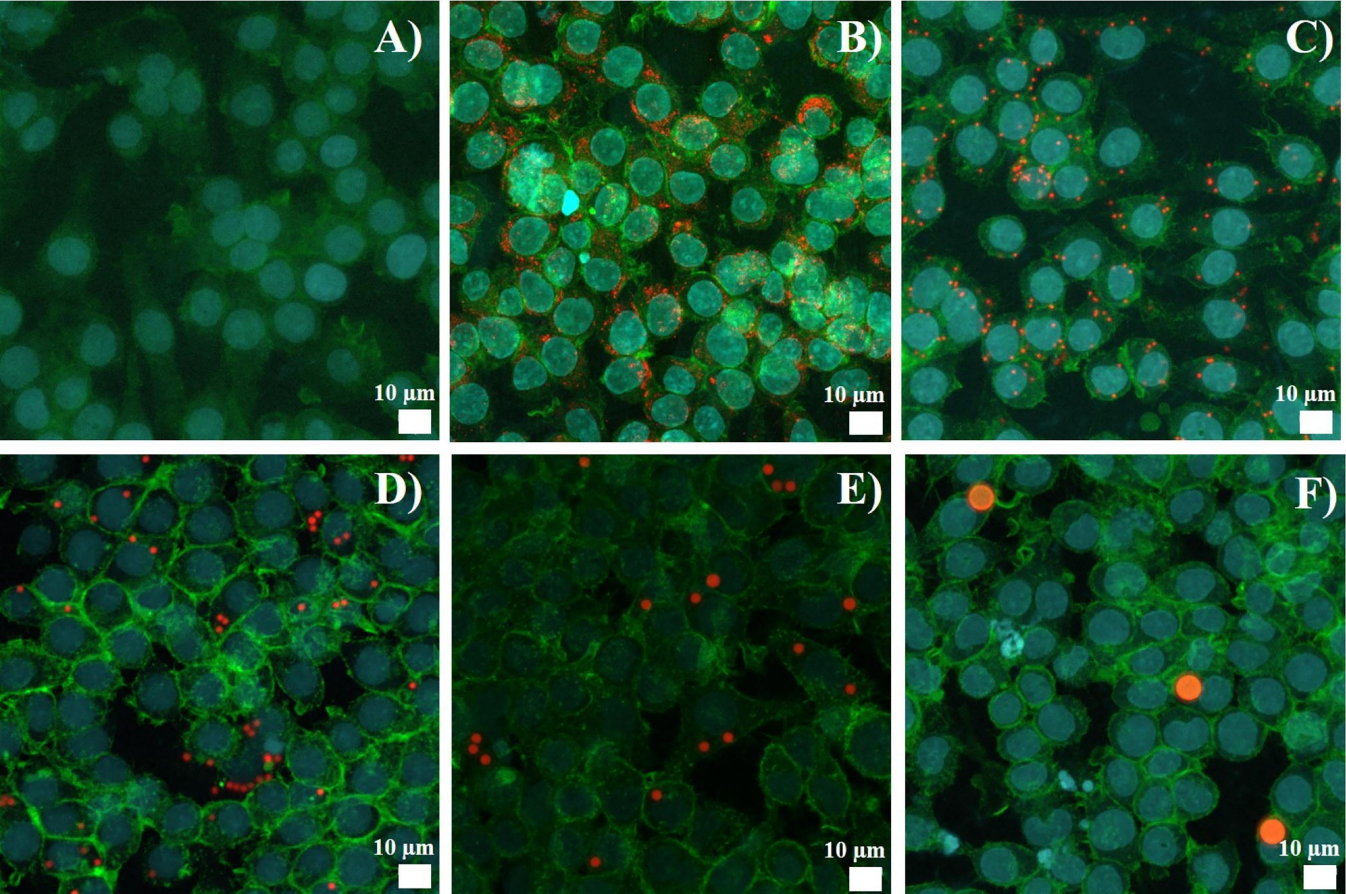
Latex bead uptake observed by confocal microscopy. J774A.1 were exposed to beads for 24 hours. Representative picture of the polystyrene beads internalization, Z-stack reconstitution. **(A)** Control cells, without bead exposure **(B)** Cells exposed to 40-90 nm beads **(C)** Cells exposed to 0.7-0.9 µm beads **(D)** Cells exposed to 1.7-2.2 µm beads **(E)** Cells exposed to 2.5-4.5 µm beads **(F)** Cells exposed to 6-8 µm beads. Nucleus is colored in blue (SytoxBlue), actin is colored in green (atto390), beads are in red (Skyblue).

As flow cytometry may detect surface-adsorbed beads as well as internalized beads, we checked this aspect by confocal microscopy. The results, shown in **Figure 1**, indicated that beads were essentially internalized.

### 3.3 Subpopulations with different particle loadings

**Figure 2:**
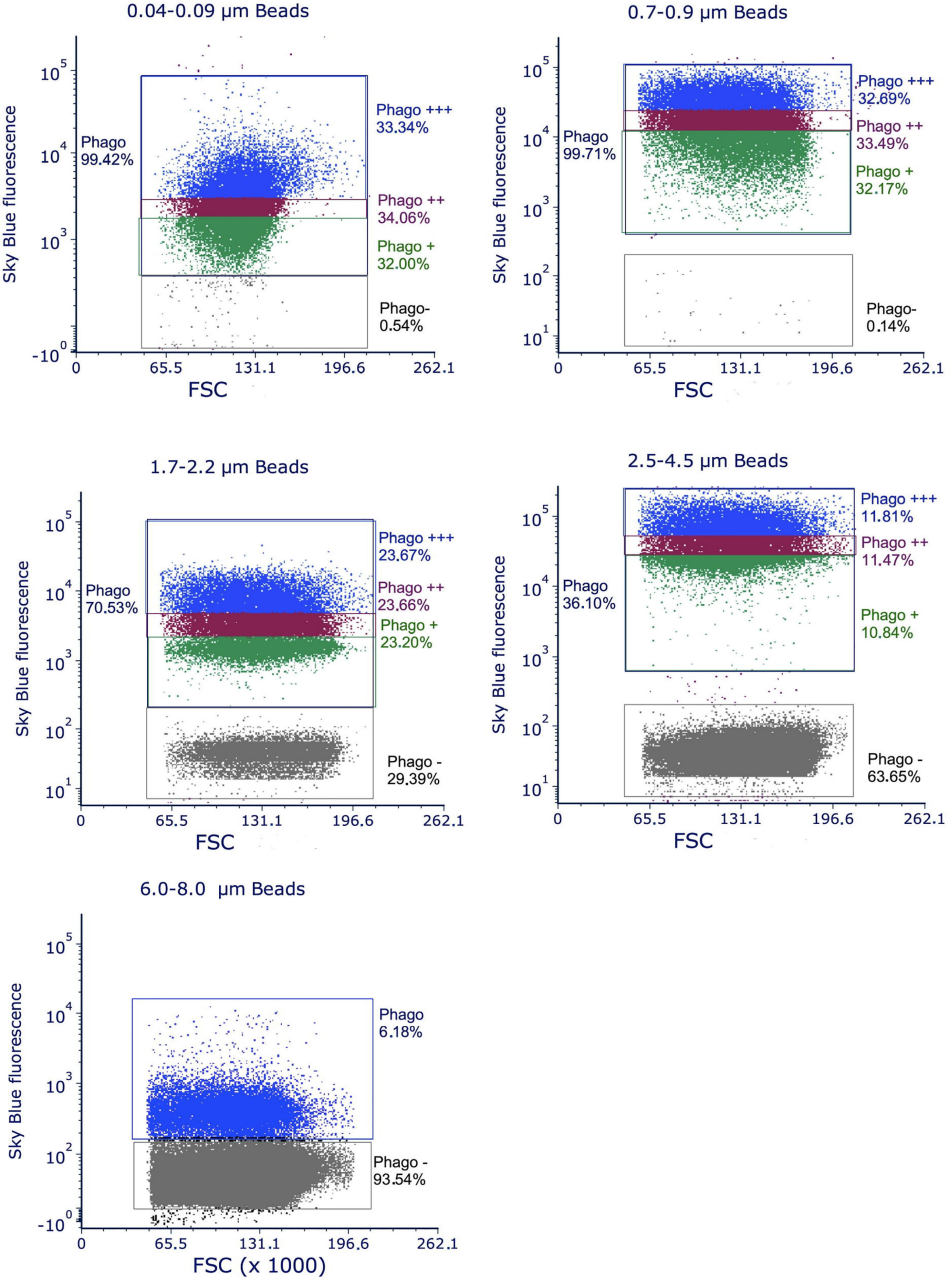
distribution of the fluorescence observed by flow cytometry on cells exposed to polystyrene beads of different sizes. The total population having internalized plastic beads is designated as Phago. It is subdivised in three thirds, graded by their fluorescence from Phago+ to Phago+++, except for the largest beads (6-8 µm), where only 7% of the cells are phagocytic and thus grouped in only one population. The distribution of the fluorescence is shown in the diagram of the figures by different colors. The population of the cells that have not internalized beads after 24 hours of exposure is designated as Phago-. The increase in this subpopulation with increasing bead size is due both to the fact that larger beads are more difficult to internalize and to the fact that at a given concentration, the number of beads decreases when beads size increases. For example, at the concentration of 20µg/ml used in the experiments, there is only 1 bead for 10 cells for 7µm diameter beads, 1.3 bead per cell for 3µm diameter beads and 4.5 beads per cell for 2µm diameter beads.

When performing the experiments with flow cytometry on bead uptake, we became aware that the distribution of the fluorescence in cells exposed to skyblue plastic particles was very wide. This suggested that after exposure the cellular population contained subpopulations differing importantly in the amount of internalized plastic particles (**Figure 2**).

This implied in turn that the physiological response of these subpopulations may also be different. To investigate this possibility, we decided to divide the population of cells having internalized plastic particles into three subpopulations of cells showing low, medium and high fluorescence after exposure to plastic particles (**Figure 2**).

This implied in turn that the physiological response of these subpopulations may also be different. To investigate this possibility, we decided to divide the population of cells having internalized plastic particles into three subpopulations of cells showing low, medium and high fluorescence after exposure to plastic particles (**Figure 2**).

These subpopulations were set to encompass one third of the phagocytic cells each. Their mean fluorescence, which is an index of the amount of plastic internalized for each subpopulation, are reported in **Table 4** for a representative experiment for each bead size.

**Table 4:** Mean Fluorescence induced by internalized plastic beads for each subpopulation.

Mean Fluorescence					
beads size	P-	Mean P	P+	P++	P+++
40-90 nm	261.4	2866.5	1264.4	2234.1	5051.9
700-900 nm	44.3	21155.9	7573.2	17927.7	38225.1
1.7-2.2 µm	43.1	4487.4	1507.9	3330.9	8563.1
2.5-4.5 µm	42.8	47922.2	22695.2	38153.4	84067.4
6-8 µm	43.2	451.3	NA	NA	451.3

NA, not applicable.

These data showed that each phagocytic subpopulation internalized ca. twice as much plastic than the previous subpopulation in the order P+<P++<P+++. This wide distribution of cells was observed for several plastic concentrations (**Supplementary Figure 3**), showing that the variability in plastic beads internalization is an intrinsic property of the wide cell population. We therefore used the lowest concentration (20µg/ml) as the one offering no toxicity while maintaining an acceptable (albeit low) proportion of phagocytic cells for large beads. We also checked by confocal microscopy that the increase in fluorescence shown by the different subpopulations corresponded to an increase in the number of internalized beads (**Supplementary Figure 4**).

### 3.4 Phagocytosis

**Figure 3:**
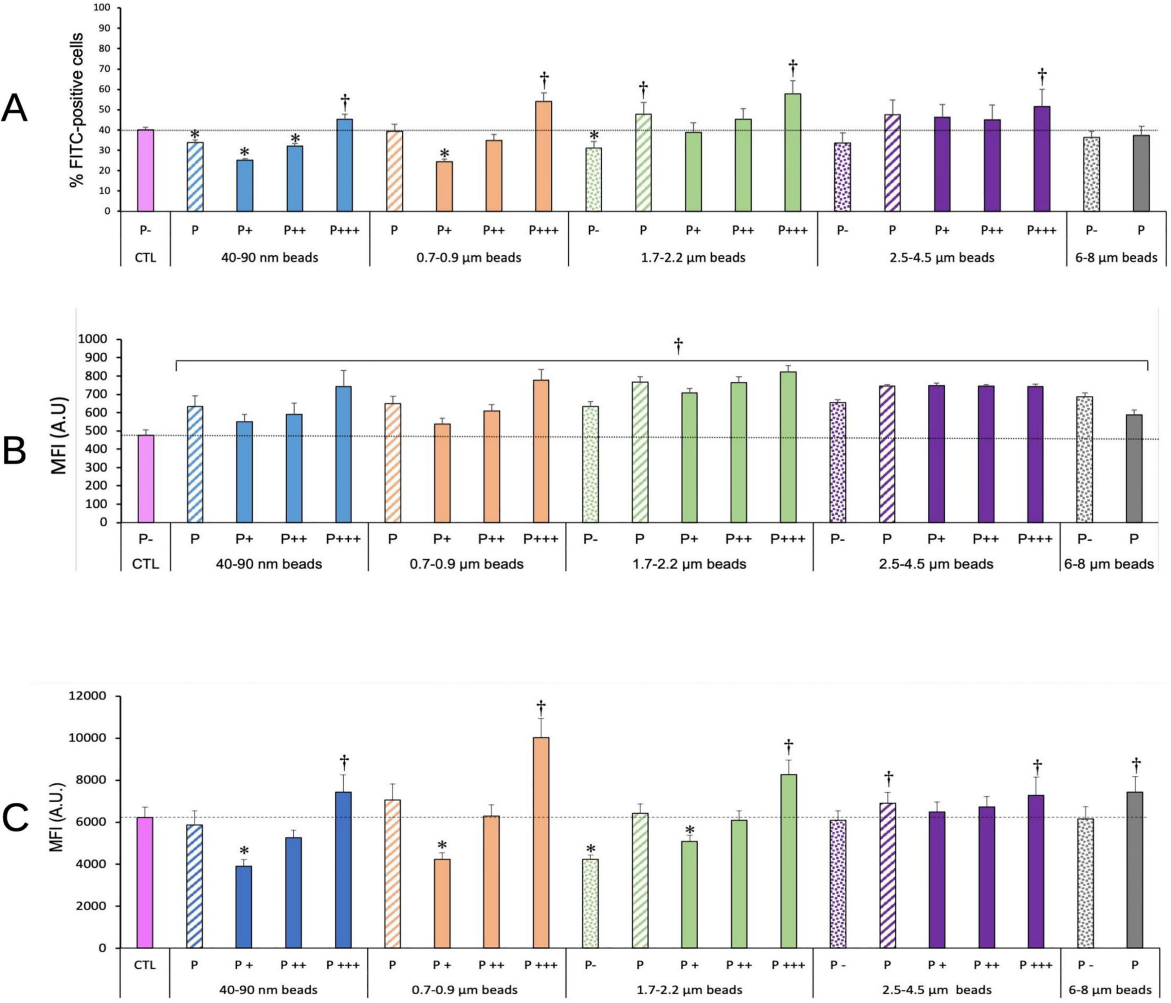
Phagocytic activity. The cells were exposed to plastic beads of different sizes for 24 hours, and finally for 3 **(A, B)** or 24 hours **(C)** to 1µm green-fluorescent beads. They were then analyzed by flow cytometry, gated for the plastic beads red fluorescence first and then analyzed for the green fluorescence. Dotted bars: cells having internalized no beads (P-). Hatched bars: total population of cells having internalized beads (P). Solid bars: subpopulations of beads-positive cells, ranked left-right from cells showing the smallest beads fluorescence (P+) to cells showing the highest beads fluorescence (P+++). Color code:Pink: cells unexposed to beads; Blue: cells exposed to 40-90 nm beads; Light orange: cells exposed to 700-900 nm beads; Green: cells exposed to 1.7-2.2 µm beads; Purple: cells exposed to 2.5-5.5 µm beads; Grey: cells exposed to 6-8 µm beads; Dotted line: value of negative control (unexposed cells); Number of experimental replicates: 5. Data are represented as mean+standard deviation. *: p<0.05 (Student T test), mean value lower than control cells. †: p<0.05 (Student T test), mean value higher than control cells. **(A)** Percentage of green fluorescence-positive cells (3 hours second exposure) **(B)** Mean fluorescence index of green fluorescence-positive cells (3 hours second exposure) **(C)** Mean fluorescence index of green fluorescence-positive cells (24 hours second exposure). All cells were positive for green fluorescence.

As the cells greatly varied in their ability to internalize the plastic beads, we checked how this internalization impacted their phagocytic ability. To this purpose, we first exposed the cells to the red-fluorescent plastic beads for 24 hours, then to green-fluorescent beads for 3 or 24 hours. At the end of this exposure scheme, we tested both the percentage of cells having internalized green beads, i.e. actively phagocytic cells, and the green fluorescence intensity of these cells, linked to the number of green beads internalized. The results, shown in **Figure 3**, showed first that the cells having internalized the largest amounts of plastic beads were also more active for phagocytosis than control, unexposed cells (**Figure 3A**), except for cells exposed to large beads (>6µm), where the activity was equivalent to the one of the control. It shall be kept in mind that this index corresponds to the proportion of cells that were able to phagocytize the green beads in only 3 hours, i.e. highly active cells.

When cells were exposed for a longer time (24 hours) to the green-fluorescent beads, thus reflecting the ability of already particles-loaded cells to internalize new beads as in a repeated exposure, it could be noticed that cells that have internalized a low number of red beads in the first round are also able to internalize a lesser amount of the green beads in the second round of exposure. Conversely, cells that have internalized a high number of red beads in the first round are also able to internalize a higher amount of the green beads in the second round of exposure. This phenomenon was also dependent on the size of the beads internalized during the first round of exposure. Cells having internalized beads in the 0.7-2.2 µm range, i.e. the size range of bacteria and yeasts, were more efficient at internalizing a second round of particles than cells having internalized beads of lower (40-90nm) or higher (>2.5 µm) size.

### 3.5 Lysosomes, mitochondria, oxidative stress

Using this subdivision strategy, we first tested general physiological parameters such as lysosomal integrity, mitochondrial transmembrane potential and oxidative stress.

**Figure 4:**
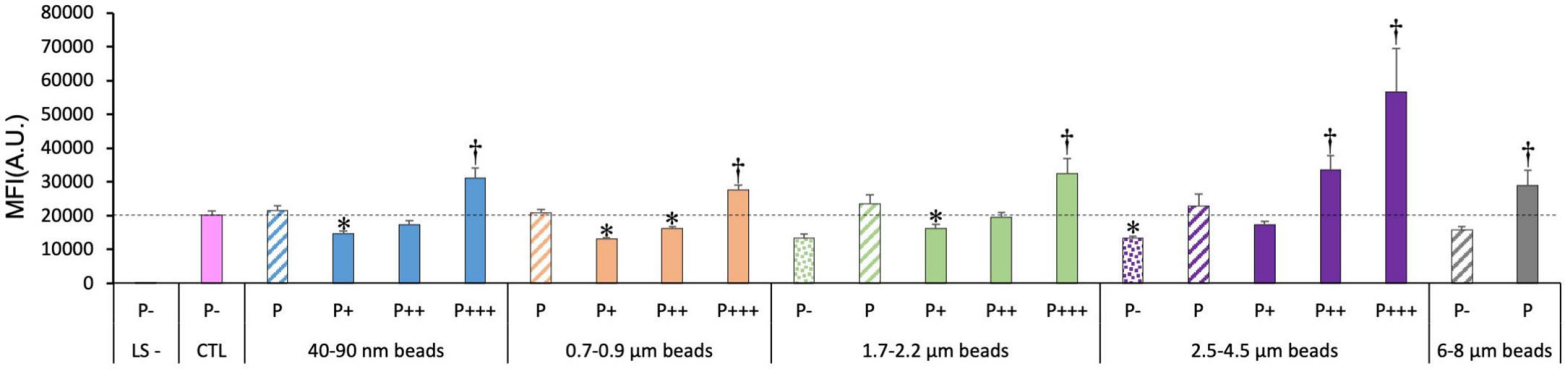
Lysosomal function, measured with the Lysosensor indicator. The cells were exposed to plastic beads of different sizes for 24 hours, and finally for 1 hour to the Lysosensor probe. They were then analyzed by flow cytometry, gated for the beads fluorescence first and then analyzed for the Lysosensor fluorescence. Dotted bars: cells having internalized no beads (P-). Hatched bars: total population of cells having internalized beads (P). Solid bars: subpopulations of beads-positive cells, ranked left-right from cells showing the smallest beads fluorescence (P+) to cells showing the highest beads fluorescence (P+++). Color code: Pink: cells unexposed to beads; Blue: cells exposed to 40-90 nm beads; Light orange: cells exposed to 700-900 nm beads; Green: cells exposed to 1.7-2.2 µm beads; Purple: cells exposed to 2.5-5.5 µm beads; Grey: cells exposed to 6-8 µm beads; Dotted line: value of negative control (unexposed cells); Number of experimental replicates: 5. Data are represented as mean+standard deviation. *: p<0.05 (Student T test), mean value lower than control cells. †: p<0.05 (Student T test), mean value higher than control cells.

Lysosomal integrity was tested with the Lysosensor probe, which senses intralysosomal pH. Thus, the fluorescence depends both on the lysosomal integrity (no leakage) and on the proper functioning of the proton ATPase to acidify the lysosomes. The results, shown in **Figure 4**, indicated clearly that the more plastic internalized, the higher the Lysosensor signal.

In more detail, it can be noticed that the weakly phagocytic cells (low bead fluorescence) and the phagocytosis-negative cells in beads-exposed cells had a lower Lysosensor signal than the control cells. The value increased to higher-than-control values in strongly phagocytic cells (high bead fluorescence) with a trend of increasing signal with increasing bead size.

For the total phagocytic population (P bars in the histograms), no significant influence of the bead size could be detected, except for the largest (6-8µm) beads, which showed a higher lysosomal signal than control cells.

**Figure 5:**
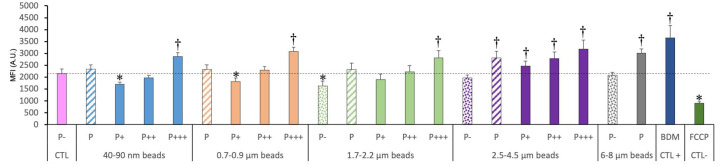
Mitochondrial transmembrane potential, measured with the Rhodamine 123 indicator. The cells were exposed to plastic beads of different sizes for 24 hours, and finally for 30 minutes to the Rh123 probe. They were then analyzed by flow cytometry, gated for the beads fluorescence first and then analyzed for the Rh123 fluorescence. Dotted bars: cells having internalized no beads (P-). Hatched bars: total population of cells having internalized beads (P). Solid bars: subpopulations of beads-positive cells, ranked left-right from cells showing the smallest beads fluorescence (P+) to cells showing the highest beads fluorescence (P+++). Color code: Pink: cells unexposed to beads; Blue: cells exposed to 40-90 nm beads.; Light orange: cells exposed to 700-900 nm beads; Green: cells exposed to 1.7-2.2 µm beads; Purple: cells exposed to 2.5-5.5 µm beads; Grey: cells exposed to 6-8 µm beads; Dark blue: cells exposed to butanedione monoxime (BDM) for 30 minutes; Dark green: cells exposed to FCCP for 30 minutes; Dotted line: value of negative control (unexposed cells); Number of experimental replicates: 5. Data are represented as mean+standard deviation. *: p<0.05 (Student T test), mean value lower than control cells. †: p<0.05 (Student T test), mean value higher than control cells.

We also tested the mitochondrial potential as a marker of the mitochondrial function. The results, shown in **Figure 5**, indicated clearly that the more plastic internalized, the higher the Rhodamine 123 fluorescence signal. As we work at low, non-quenching concentration, a higher signal indicated a higher transmembrane potential. This interpretation was confirmed by the signals obtained with the mitochondrial inhibitors, FCCP (a proton decoupler) inducing a lower transmembrane potential and a lower Rhodamine 123 signal.

For the total phagocytic population (P bars in the histograms), an influence of the bead size could be detected, but only for large (>2.5µm) beads, which showed a higher signal than control cells.

In more detail on the different subpopulations, it can be noticed that the weakly phagocytic cells (low bead fluorescence) and the phagocytosis-negative cells in beads-exposed cells had a lower Rh123 signal than the control cells, but only for low-size beads (lesser than 1 micron). The value increased to higher-than-control values in strongly phagocytic cells (high bead fluorescence) with a trend of increasing signal with increasing bead size, reaching values close to the ones of the positive control (cells treated with butanedione monoxime).

**Figure 6:**
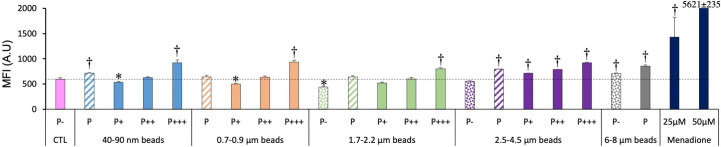
Cellular oxidative stress, measured with the dihydrorhodamine 123 (DHR123) indicator. The cells were exposed to plastic beads of different sizes for 24 hours, and finally for 30 minutes to the Rh123 probe. They were then analyzed by flow cytometry, gated for the beads fluorescence first and then analyzed for the Rh123 fluorescence. Dotted bars: cells having internalized no beads (P-). Hatched bars: total population of cells having internalized beads (P). Solid bars: subpopulations of beads-positive cells, ranked left-right from cells showing the smallest beads fluorescence (P+) to cells showing the highest beads fluorescence (P+++). Color code: Pink: cells unexposed to beads; Blue: cells exposed to 40-90 nm beads; Light orange: cells exposed to 700-900 nm beads; Green: cells exposed to 1.7-2.2 µm beads; Purple: cells exposed to 2.5-5.5 µm beads; Grey: cells exposed to 6-8 µm beads; Dark blue: cells exposed to menadione for 2 hours; Dotted line: value of negative control (unexposed cells); Number of experimental replicates: 5. Data are represented as mean+standard deviation. *: p<0.05 (Student T test), mean value lower than control cells. †: p<0.05 (Student T test), mean value higher than control cells.

We then tested the oxidative stress response of the cells exposed to plastic beads. The results, shown in **Figure 6**, indicated clearly that the more plastic internalized, the higher the DHR123 signal. For the total phagocytic population (P bars in the histograms), it could be noted that both the very small (40-90 nm) and the large beads (>2.5µm) induced a higher oxidative stress signal that in control cells, while on average, medium size beads (0.7-2.2 µm) did not show this effect.

In more detail on the different subpopulations, it can be noticed that the simple presence of the largest beads (6-8µm) in the culture well is sufficient to induce a weak but significant oxidative stress. The weakly phagocytic cells (low bead fluorescence), and the phagocytosis-negative cells in 1.7-2.2 µm beads-exposed cells showed a lower DHR123 signal than the unexposed cells. Conversely, the strongly phagocytic cells (high bead fluorescence) showed a DHR123 signal higher than unexposed cells (1.5 fold on average), to be compared with the 2.4 fold increase in DHR123 signal induced by a treatment with 25µM menadione and the 10 fold increase induced by a treatment with 50µM menadione.

### 3.6 Surface markers

We the tested more specific responses of macrophages to polystyrene beads of different sizes through the analysis of surface markers. For surface markers, two aspects were tested. First the percentage of cells that were positive for the antigen examined, and then the intensity of the labelling for positive cells, through the Mean Fluorescence Index (MFI).

#### 3.6.1 Integrins

**Figure 7:**
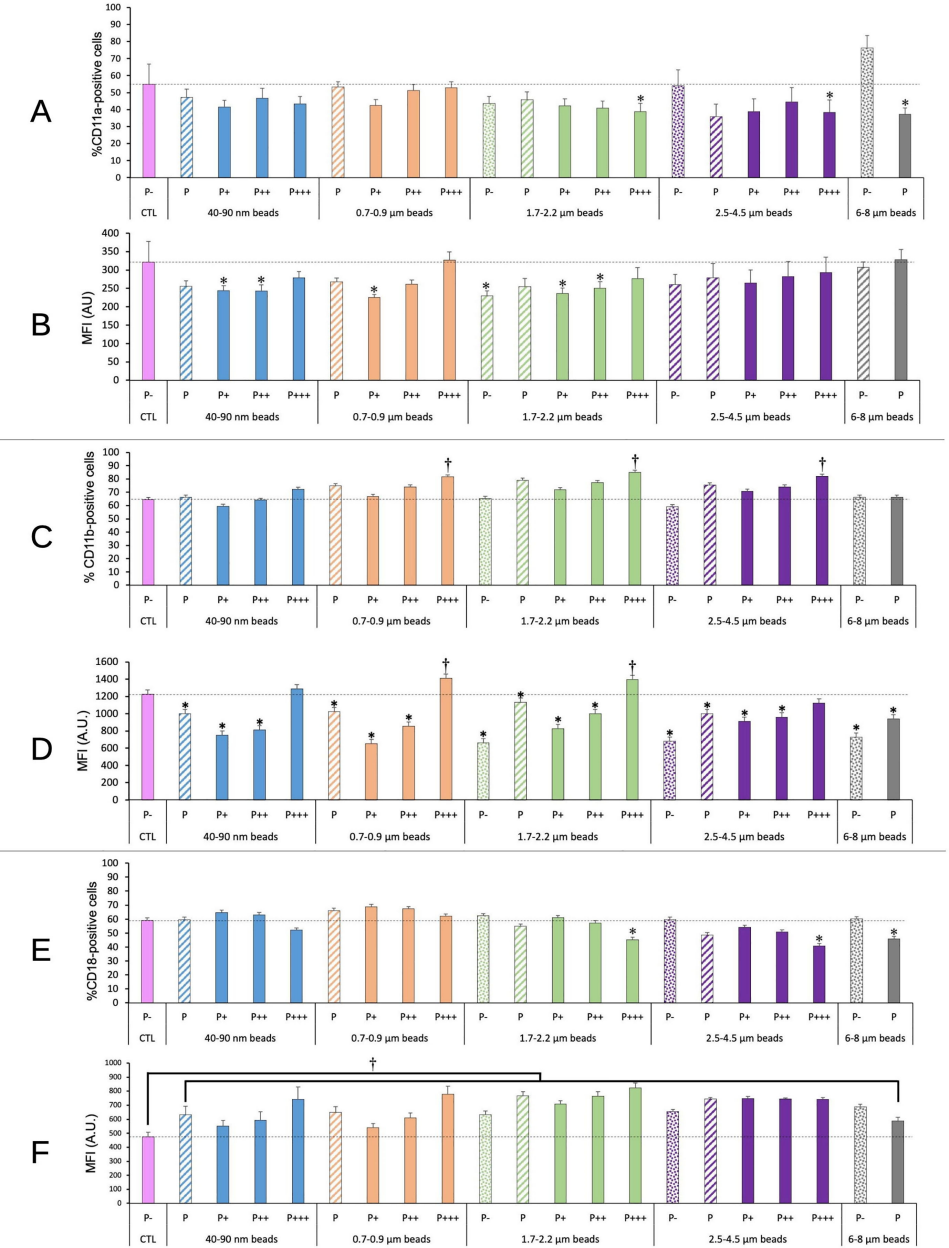
CD11a/CD11b/CD18 surface expression. The cells were exposed to plastic beads of different sizes for 24 hours, and finally tested for the surface expression of the CD11a/CD11b/CD18 integrins. They were then analyzed by flow cytometry, gated for the beads fluorescence first and then analyzed for the conjugated antibodies fluorescence. Dotted bars: cells having internalized no beads (P-). Hatched bars: total population of cells having internalized beads (P). Solid bars: subpopulations of beads-positive cells, ranked left-right from cells showing the smallest beads fluorescence (P+) to cells showing the highest beads fluorescence (P+++). Color code: Pink: cells unexposed to beads; Blue: cells exposed to 40-90 nm beads; Light orange: cells exposed to 700-900 nm beads; Green: cells exposed to 1.7-2.2 µm beads; Purple: cells exposed to 2.5-5.5 µm beads; Grey: cells exposed to 6-8 µm beads; Dotted line: value of negative control (unexposed cells); Number of experimental replicates: 5. Data are represented as mean+standard deviation. *: p<0.05 (Student T test), mean value lower than control cells. †: p<0.05 (Student T test), mean value higher than control cells. **(A)** Percentage of CD11a-positive cells **(B)** Mean fluorescence index of CD11a-positive cells **(C)** Percentage of CD11b-positive cells **(D)** Mean fluorescence index of CD11b-positive cells **(E)** Percentage of CD18-positive cells **(F)** Mean fluorescence index of CD18-positive cells.

We tested the integrins CD11a, CD11b and CD18. First as integrins they contribute to the adhesion of macrophages. Second the CD11b/CD18 dimer forms complement receptor 3, while the CD11a/CD18 dimer forms the LFA antigen. The results, shown on **Figure 7**, indicated a small (-25-30%) but statistically significant decrease in the proportion of CD11a and CD18 only for highly fluorescent cells exposed to micrometric (1.7-4.5µm) beads, and also for CD11a for cells having phagocytized large (6-8 µm) beads. Conversely, a small (+25-30%) but statistically significant increase was observed in the proportion of CD11b-positive cells for highly fluorescent cells exposed to submicrometric and micrometric (0.7-4.5µm) beads, but neither for nanometric beads nor for large (>6µm) beads.

Regarding the labelling intensity, reflecting the amount of antigen detectable on the cell surface, a small (-20-25%) and sometimes statistically significant decrease was observed in CD11a and CD11b levels for cells showing low or intermediate beads fluorescence, while the cells showing high beads fluorescence showed CD11a and CD11b levels almost identical or slightly higher (+15%) to those of control cells (for CD11b). CD18 levels were always increased in plastic beads-treated cells compared to control ones, the increase reaching +50% for the cells showing high beads fluorescence. When taking into account the global phagocytic population (P bars in the histograms), no significant change could be detected on the proportion of CD11a, CD11b or CD18-positive cells. A slight significant decrease in the labelling intensity of CD11b-positive cells could be detected, but was close to constant over the entire beads size range. The situation was different for CD18. All the beads induced an increase in the surface labelling for CD18. However, this increase was more pronounced for beads in the 1.7-4.5 range than for smaller (40-900 nm) and larger (6-8 µm) beads. When compared to the labelling intensity shown by cells exposed to 40-90 nm beads, cells exposed to 1.7-2.2 µm beads and cells exposed to 2.5-4.5µm beads showed a significantly (p>0.05 in Student T test) higher (respective MFI ratio 1.21 and 1.18) labelling intensity. This trend was produced by the low fluorescence population, which surface labelling increased respectively by a factor of 1.28 and 1.35 compared to the one showed by the low fluorescence population in cells exposed to 40-90 nm beads.

#### 3.6.2 Antigen presentation

**Figure 8:**
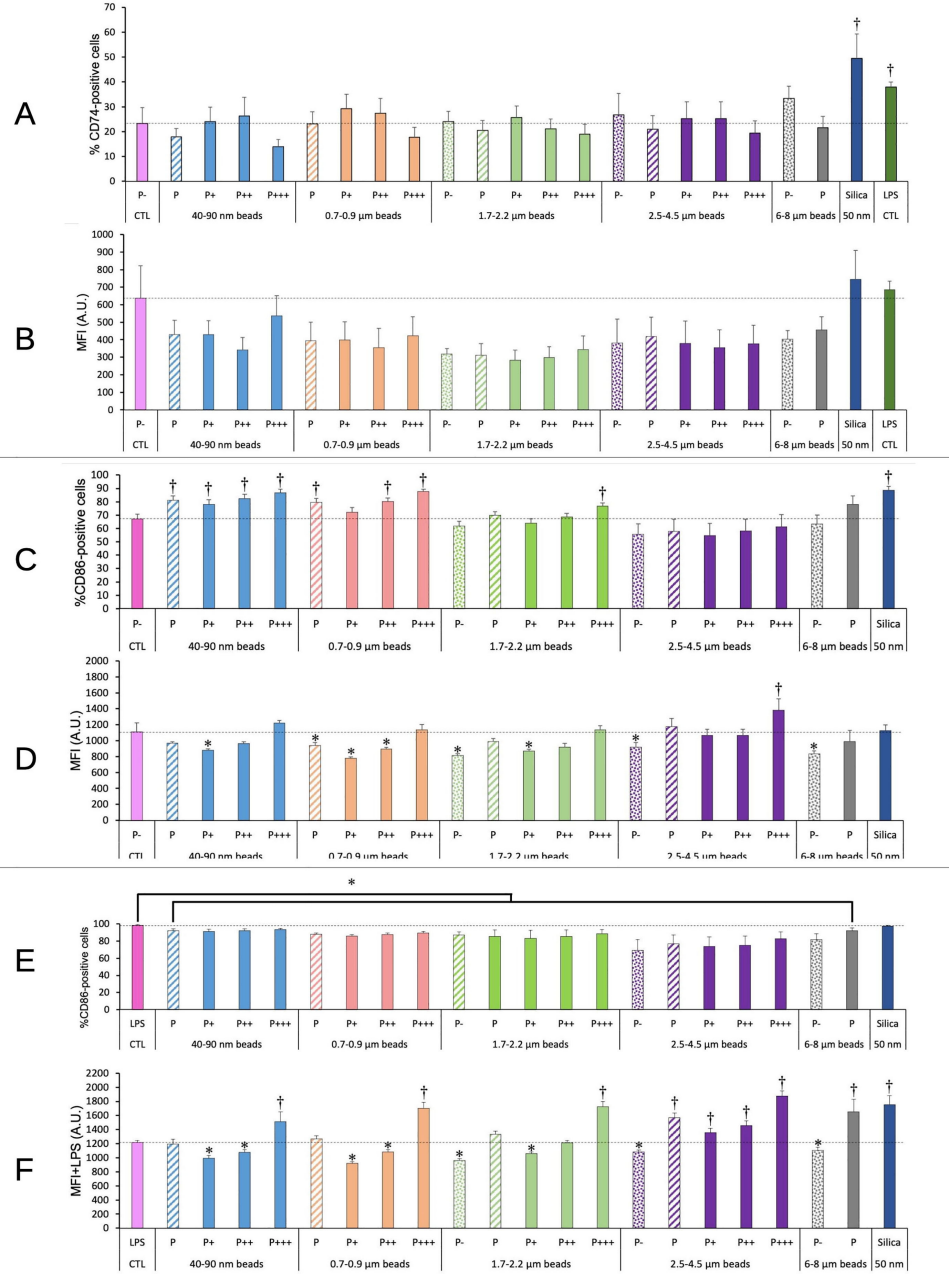
CD74/CD86 surface expression. The cells were exposed to plastic beads of different sizes for 24 hours, and finally tested for the surface expression of CD74 and CD86. They were then analyzed by flow cytometry, gated for the beads fluorescence first and then analyzed for the conjugated antibodies fluorescence. Dotted bars: cells having internalized no beads (P-). Hatched bars: total population of cells having internalized beads (P). Solid bars: subpopulations of beads-positive cells, ranked left-right from cells showing the smallest beads fluorescence (P+) to cells showing the highest beads fluorescence (P+++). Color code: Pink: cells unexposed to beads; Blue: cells exposed to 40-90 nm beads; Light orange: cells exposed to 700-900 nm beads; Green: cells exposed to 1.7-2.2 µm beads; Purple: cells exposed to 2.5-5.5 µm beads; Grey: cells exposed to 6-8 µm beads; Dark blue: cells exposed to colloidal silica; Dark green: cells exposed to LPS; Dotted line: value of negative control (unexposed cells); Number of experimental replicates: 5. Data are represented as mean+standard deviation. *: p<0.05 (Student T test), mean value lower than control cells. †: p<0.05 (Student T test), mean value higher than control cells. **(A)** Percentage of CD74-positive cells **(B)** Mean fluorescence index of CD74-positive cells **(C)** Percentage of CD86-positive cells **(D)** Mean fluorescence index of CD86-positive cells **(E)** Percentage of CD86-positive cells when treated with LPS and plastic particles **(F)** Mean fluorescence index of CD86-positive cells when treated with LPS and plastic particles.

As we observed modulations in cell surface molecules involved in antigen presentation (e.g. CD86) in cells exposed to amorphous silica (41), we decided to test such molecules in the response to plastic particles. The results, shown on **Figure 8**, indicated no significant change for CD74, i.e. the invariant chain of MHC2, neither in the percentage of CD74-positive cells nor in the MFI of surface CD74.

For CD 86, the plastic particles alone induced an increase in the percentage of CD86-positive cells for small beads (<1µm), which disappeared for larger beads and seemed to reappear for very large beads (>6µm, although at a statistically not significant level (p=0.06). Regarding the MFI of the CD86-positive cells, it was decreased by 20-30% for poorly phagocytic and non-phagocytic cells, and here again the effect was less pronounced for larger beads.

When the cells were co-treated with LPS and plastic beads, in order to mimic a bacterial challenge occurring together with the exposure to plastics, an increase in the proportion of CD86-positive cells was observed for cells treated with LPS alone. Because this percentage was very high and very stable (98 ± 1%), the 10% decrease observed when cells were co-treated with LPS and plastic beads appeared statistically significant. The MFI of the positive cells was slightly reduced for non-phagocytic cells and for poorly phagocytic cells, and increased above the control level for highly phagocytic cells. In general, the MFI for CD86 in cells co-treated with LPS and plastic beads increased when the beads size increased.

#### 3.6.3 PAMPS/DAMPS

**Figure 9:**
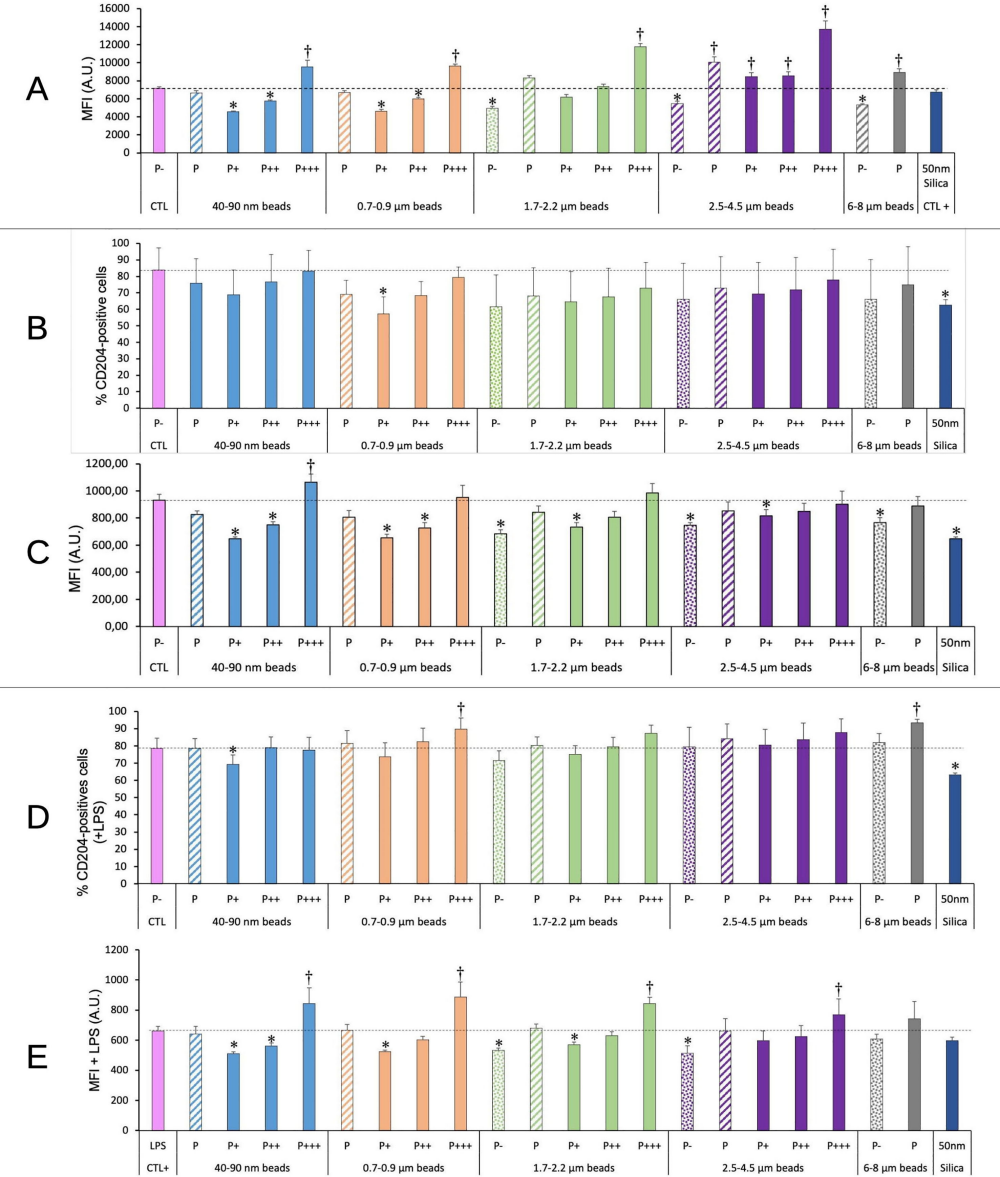
TLR7/CD204 surface expression. The cells were exposed to plastic beads of different sizes for 24 hours, and finally tested for the expression of TLR7 and the surface expression of CD204. They were then analyzed by flow cytometry, gated for the beads fluorescence first and then analyzed for the conjugated antibodies fluorescence. Dotted bars: cells having internalized no beads (P-). Hatched bars: total population of cells having internalized beads (P). Solid bars: subpopulations of beads-positive cells, ranked left-right from cells showing the smallest beads fluorescence (P+) to cells showing the highest beads fluorescence (P+++). Color code: Pink: cells unexposed to beads; Blue: cells exposed to 40-90 nm beads; Light orange: cells exposed to 700-900 nm beads; Green: cells exposed to 1.7-2.2 µm beads; Purple: cells exposed to 2.5-5.5 µm beads; Grey: cells exposed to 6-8 µm beads; Dark blue: cells exposed to colloidal silica; Dotted line: value of negative control (unexposed cells); Number of experimental replicates: 5. Data are represented as mean+standard deviation. *: p<0.05 (Student T test), mean value lower than control cells. †: p<0.05 (Student T test), mean value higher than control cells. **(A)** Mean fluorescence index of cells for TLR7 (all cells were positive) **(B)** Percentage of CD204-positive cells **(C)** Mean fluorescence index of CD204-positive cells **(D)** Percentage of CD204-positive cells when treated with LPS and plastic particles **(E)** Mean fluorescence index of CD204-positive cells when treated with LPS and plastic particles.

As we observed modulations in some cell surface molecules involved in molecular patterns recognitions (e.g. TLR7 or CD204) upon exposure to polystyrene beads through a proteomic screen (25), we investigated this phenomenon into more detail. The results, shown on **Figure 9**, indicated an increase in the TLR7 fluorescence for highly phagocytic cells, and this increase propagated to lesser phagocytic cells for large beads (>3µm). A clear influence of the beads size could be detected on the global phagocytic cell population (P bars in the histograms). An increase was noted for beads higher than 1µm in diameter, which became statistically significant for large (>2.5µm) beads.

For CD 204, the high variability of the percentage of positive cells made the results difficult to interpret. However, the intensity of fluorescence (MFI) decreased by 20-30% for non-phagocytic and poorly phagocytic cells, and increased to above control values (by 15%) only for highly phagocytic cells and small nanoparticles. The fluorescence intensity also decreased by 30% for cells exposed to nanosilica (positive effect control). No significant change could be detected at the level of the global phagocytic population.

For cells co-treated with plastic beads and LPS, the same type of response was observed. In this case, however, highly phagocytic cells reached MFI values higher than control ones by 20-30% for all beads sizes.

#### 3.6.4 PD-L1

**Figure 10:**
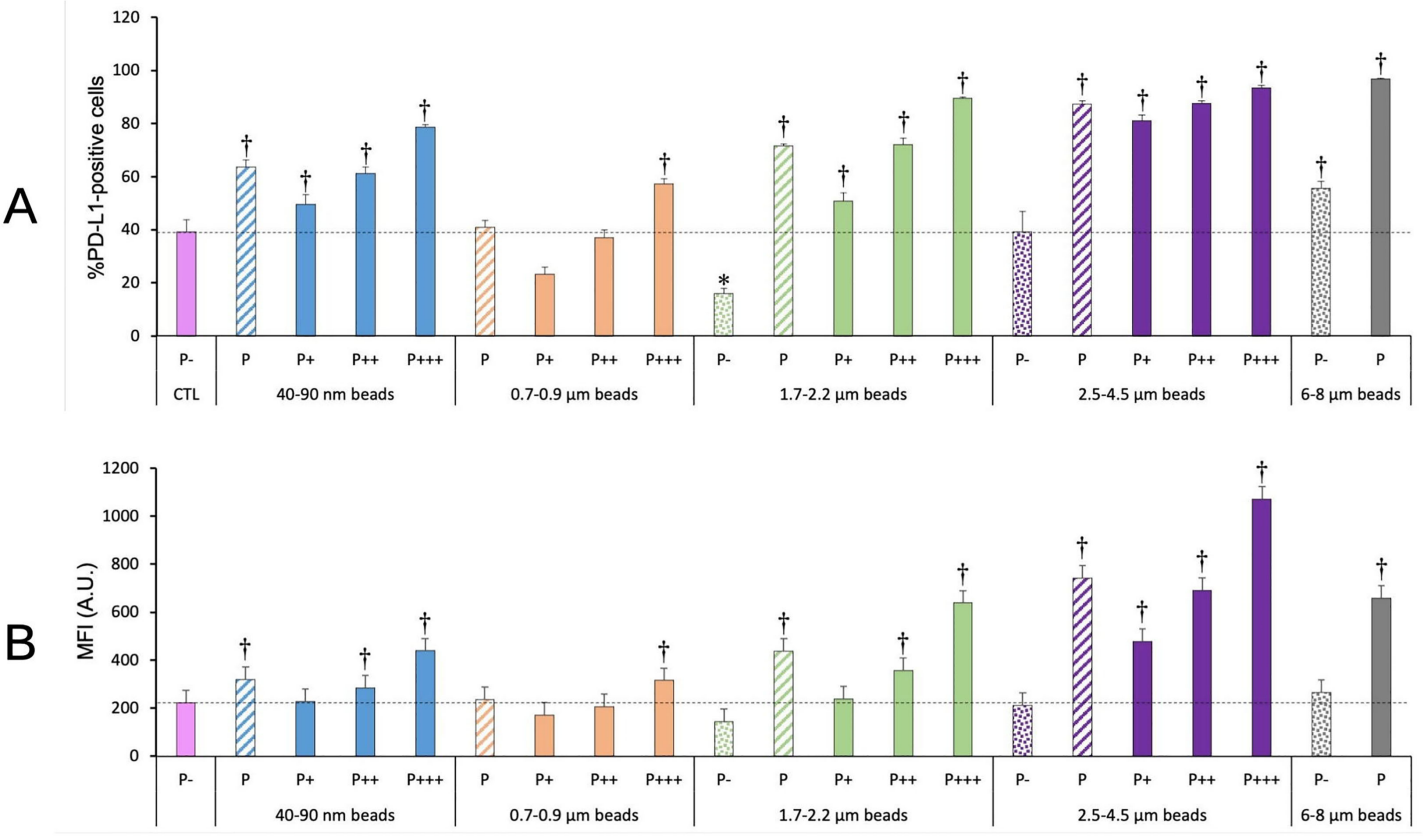
PD-L1 surface expression. The cells were exposed to plastic beads of different sizes for 24 hours, and finally tested for the surface expression of PD-L1. They were then analyzed by flow cytometry, gated for the beads fluorescence first and then analyzed for the conjugated antibodies fluorescence. Dotted bars: cells having internalized no beads (P-). Hatched bars: total population of cells having internalized beads (P). Solid bars: subpopulations of beads-positive cells, ranked left-right from cells showing the smallest beads fluorescence (P+) to cells showing the highest beads fluorescence (P+++). Color code:Pink: cells unexposed to beads; Blue: cells exposed to 40-90 nm beads; Light orange: cells exposed to 700-900 nm beads; Green: cells exposed to 1.7-2.2 µm beads; Purple: cells exposed to 2.5-5.5 µm beads; Grey: cells exposed to 6-8 µm beads; Dotted line: value of negative control (unexposed cells); Number of experimental replicates: 5. Data are represented as mean+standard deviation. *: p<0.05 (Student T test), mean value lower than control cells. †: p<0.05 (Student T test), mean value higher than control cells. **(A)** Percentage of PL-L1-positive cells **(B)** Mean fluorescence index of PD-L1-positive cells.

The PD-L1 ligand, expressed at the surface of macrophages among other cell types (42), plays an important role in the control of the immune response (43). We thus decided to investigate if and how it was modulated in response to plastic beads. The results, shown on **Figure 10**, indicated an increase in both the percentage of PD-L1 positive cells and of the fluorescence intensity of the positive cells (MFI), except for cells exposed to 0.7-0.9 µm particles, for which an increase was observed only for the most phagocytic cells. The response appeared correlated with the amount of plastic internalized, as it was dependent on both the beads size and on the phagocytic index, as measured from the fluorescence of the internalized beads.

When taking into account the total phagocytic population (P bars in the histograms), a clear U-shape curve could be detected at both levels (percentage of positive cells and extent of surface labelling). Both small (40-90 nm) and large (>1.7µm) particles showed higher signals than control cells, and the signals increased with particle size, with a small final decrease for very large (6-8µm) particles

## 4 Discussion

Because of their industrial activities (e.g. mining, construction work), lifestyle (e.g. tattooing) and the pollution that they generate (e.g. fossil fuels combustion, plastic pollution) humans (among other species) are more and more exposed to particulate materials. In the body, professional phagocytes of the innate immune system and among them macrophages, are at the frontline to cope with these particulate materials (e.g (27, 44–46).). Even if the immune system is known to be resilient, coping with particulate materials can lead to pathological conditions through various mechanisms, such as overload (e.g (47, 48).) or chronic inflammation such as in silicosis or asbestosis (e.g (49, 50). It is thus quite important to understand how macrophages react to the various types of particulate materials that they may encounter.

Compared to soluble substances, particulate materials offer a number of peculiarities. Because of their size, they cannot diffuse easily, so that local/gradient effects are more pronounced than for soluble substances. In the same trend, because they cannot enter passively into cells, they are actively taken up by various cellular processes such as pinocytosis or phagocytosis. These peculiarities, combined with the intrinsic variabilities that always exist in cellular populations, lead to the concept that a population of cells exposed to a particulate material may finally produce subpopulations that have internalized variable amounts of particles. This may explain the focal presentation of particles-associated pathologies such as in silicosis (49, 51).

We thus decided to investigate if such subpopulations may exist when a population of macrophages is exposed to nano- or microplastics of different sizes, and if these subpopulations would show different phenotypes in response to these various concentrations of internalized plastics. The data obtained by flow cytometry showed (i) that the population of cells having internalized fluorescent plastic beads was widely distributed in the fluorescence signal, (ii) that this distribution decreased in amplitude when the size of the beads increased, and (iii) that the splitting of the population in three equally populated subpopulations showed a narrow median zone and two more dilated extreme zones, indicating a gaussian (peak)-like distribution. All these features are consistent with a model where particles are unevenly distributed. As the size of the particles decreases, the ease of internalization increases and the number of particles available per cell (at a fixed mass concentration) increases. This explains why the spreading of the fluorescence is higher for small beads than for larger ones.

Once this subpopulation sorting scheme was validated, we first investigated general cellular parameters, in relation with what is already known for plastic particles or for other specific particulate materials such as silica. For example, we tested part of the lysosomal function with the Lysosensor probe. As this probe is pH sensitive (more fluorescent with a lower pH), this tested the amount of functional lysosomes, i.e. intact lysosomes with an active proton pump. The results were very logical, with non-phagocytic and poorly phagocytic cells showing a lower lysosomal activity than control (untreated) cells and highly phagocytic cells showing a higher lysosomal activity than control cells. Interestingly, the fluorescence value increased for larger (>3µm) beads, and decreased for very large beads (>6µm) were only one or two beads can be internalized by a single cell.

We then tested the mitochondrial transmembrane potential, as an indicator of the general oxidative metabolism. We observed a slightly reduced potential (-20% compared to control) for poorly phagocytic cells, but this reduction was low compared to the reduction produced by a true uncoupler such as FCCP (-60%). Interestingly, we observed an increased mitochondrial potential for highly phagocytic beads, generalizing a previous observation made on large polystyrene beads (25). This increase was more present for larger beads, as it was observed for all phagocytic cells for beads larger than 3µm. It was also comparable in intensity to the increased brought by a classical positive compound (butanedione monoxime) (39). This is an important observation, as an increased mitochondrial transmembrane potential is known to correlate with an increased oxidative stress (52, 53).

This prompted us to investigate the oxidative stress induced in macrophages by plastic particles. The trend paralleled the one observed for the mitochondrial potential, with highly phagocytic cells and cells exposed to larger beads showing an increased level of reactive oxygen species. The levels reached (x1.3-1.5 fold compared to control cells) were lower than those reached for the positive control (x2.4-9.4 fold), but it should be kept in mind that such levels are lethal to cells in a few hours, while the cells exposed to plastics have survived a 24 hours exposure to the plastic beads. An increase in reactive oxygen species has also been observed for carboxypolystyrene (22), while we used here plain polystyrene. An increase in reactive oxygen species has also been described for very small plain polystyrene nanoparticles (40nm) (23).

Macrophages being exceptionally resistant to oxidative stress, this increase may prove not deleterious to macrophages themselves. However, it is also known that hydrogen peroxide can be detected extracellularly in inflammation (54, 55). This extracellular hydrogen peroxide may impact in turn the surrounding tissue, as known for silicosis (56). As nanoplastics have been detected in lungs (6), this oxidative stress may prove a point of attention. Similarly, as plastic particles have been shown to translocate through the intestinal barrier (10, 11), they are likely to be captured by the intestinal macrophages. The ensuing oxidative stress may play a role in the observed and reactive oxygen species-dependent intestinal dysfunction observed after plastic ingestion (57). Finally for this point, the oxidative stress appears to be plastic dose-dependent. As we have shown here that plastic particles are not eliminated from macrophages over time, the final immune effects may be dependent on the overall time of exposure to plastics (and thus on the final plastic dose).

We then tested a panel of various surface markers, chosen on the basis of previous observations made on other particles types. For example, in the case of tattooing, pigmented cells were observed in lymph nodes (58), leading to the suspicion that pigments loaded macrophages may migrate from the tattoo site to the draining lymph node. As this migration may be linked with the integrin status of the macrophages, which has been found to be altered in pigments-loaded macrophages (30), we probed the surface level of the integrins of the CD11/CD18 family. We did not observe large effects in the proportion of CD11/CD18 positive cells after exposure to plastic particles. However, the results need to be commented in detail.

When paired with CD18, CD11b forms complement receptor 3 (59), and plays a role in innate immunity though various mechanisms (e.g. in (60, 61)). We observed that cells having internalized large amounts of beads in the 0.7-4.5µm range showed a higher proportion of CD11b-positive cells and a higher surface expression of this marker. The effects were subtle (+25-30 in the proportion of positive cells, +15% in the surface expression) but we do not know what range of change is relevant in an *in vivo* context. Interestingly, cells of low phagocytic activity showed a reduced surface expression of CD11b (-25-50%). These contrasting effects suggest that different perturbations of the immune system may occur depending on the total plastic loading of the cells and on the plastic particles sizes.

In addition, the proportion of CD18-negative cells increases from 40% in control cells to 54-59% for the highly phagocytic cells and for large particles (>1.5µm) only. This result is further reinforced by the results obtained on CD11a, which forms the LFA-1 integrin when paired with CD18 (62). As for CD18, the proportion of CD11a-negative cells increased from 40% in control cells ca. 60% for the highly phagocytic cells and for large particles (>1.5µm) only.

Moreover, a decrease in the surface expression of CD11a (-20-30%) was observed for CD11a-positive cells having phagocytized a moderate amount of beads, while for the cell population detected as CD18-positive, exposure to plastic beads lead to a small but significant increase (+15-70%) in CD18 surface expression, as previously described for amorphous silica (41).

As CD18 is able to pair with several alpha integrin chains, these results suggest a slightly lower surface expression of the LFA-1 integrin on cells having internalized plastic beads. As LFA-a is involved in T cell activation (63), these results suggest a lowered ability of plastic loaded cells to activate T lymphocytes.

This conclusion is further reinforced by the data obtained on PD-L1. Both the proportion of PD-L1-positive cells and the surface expression of PD-L1 were increased for cells having internalized a high amount of plastic beads, either as a high number of small beads or as larger beads, even in a smaller number. As PD-L1 is known to slow down T cell responses (43), it appears that all our data converge in shaping a picture where plastic-loaded macrophages are less able to stimulate T cells, with a plastic dose-dependent response.

Beside their general functions on T cell activation, macrophages can also interact with T cells through their antigen presentation function. Previous work on antigen-presenting cells such as dendritic cells have shown that some nanomaterials, such as silica, are able to activate dendritic cells by themselves (64, 65). it was therefore of interest to determine whether a similar situation was encountered with plastic particles. We thus investigated the surface expression of CD74, i.e. the invariant chain of MHC2, and of the co-activator CD86. In these experiments and based on the knowledge existing on dendritic cells, we used nanosilica as a positive control. Indeed silica increased the proportion of CD74-positive cells and the one of CD86-positive cells. However, the proportion of CD74-positive cells was not significantly modified in response to plastic particles. In contrast, the proportion of CD86-positive cells was slightly increased, but only for cells having internalized low-size beads (sub-micron) or for the most phagocytic cells having internalized beads of ca. 2µm. Internalization of larger beads did not produce this effect. The surface expression of CD86 for positive cells followed a complex trend, in which a slight decrease was observed for cells having internalized a limited amount of plastics (i.e. low phagocytic cells and small plastic beads), while the value increased for cells having internalized larger beads and was higher than the control value for the most phagocytic cells and large (3µm) beads. This represented the basal state induced by plastic beads internalization, but we decided to investigate the interference of the ingestion of plastic beads with the response induced by bacterial compounds, e.g. LPS. As expected, LPS induced a major increase in the proportion of CD86-positive cells, from 66% to 98%. This proportion was slightly (but statistically significantly) lowered in the case of co-exposure to plastics and LPS, but the decrease was minimal (from 98% to ca. 90% for sub-beads up to 2µm, and 80% for 3µm beads). In this co-exposure scheme, the surface expression of CD86 followed the same trend than without LPS, but with higher values, so that the most phagocytic cells showed a higher expression (+25-50%) than in the control cells, reaching the same values than those obtained for silica-LPS co-exposure.

Finally, we also investigated some of the PAMPS/DAMPS surface receptors. We focused on TLR7, as we found it increased at the proteomic level in our previous work on polystyrene particles (25), but also on CD204, also known as Scavenger Receptor 1. CD204 is known to have a wide scope of anionic ligands (66), and it is likely to be involved in the massive particle uptake (67) and activation processes demonstrated with strongly negatively charged plastic particles on macrophages (22, 24, 68) and dendritic cells (69).

As TLR7 is mostly intracellular (70), we did not focus on its surface expression but on its overall expression in cells. All cells were positive for TLR7 expression, so that we focused on its quantitative expression. Once again the value seemed to depend on the amount of internalized plastics: lower than the control for non or moderately phagocytic cells and small particles (sub-micron), higher than the control (+30-90%) for highly phagocytic cells and/or large plastic beads. As the interaction of TLR7 with its endogenous RNA ligands has been shown to play a role in arthritis (71, 72), this result suggests that cells heavily loaded with plastic particles may become able to generate inflammatory responses more easily upon secondary stimulation with TLR7 ligands and thus favor this type of inflammatory responses. Indeed, prolonged arthritic responses have been observed in mice exposed orally to microplastics (73), in line with our results.

Although CD 204 preferentially binds strongly anionic particles, the polystyrene particles that we used showed a negative surface potential and thus may behave as CD204 ligands, justifying to investigate this particular molecule, which is also considered as a marker of the M2 macrophage polarization state (20).

First, we investigated the evolution of the surface expression in response to plastic beads alone. The percentage of CD204-positive cells was quite variable from one culture to another, so that statistically significant results could not be obtained. The fluorescence value for positive cells was however much more stable, and led to the classical scheme of lower-than-control values for poorly phagocytic cells, increasing for cells with a higher phagocytosis. Nevertheless, only highly phagocytic cells exposed to nano beads (70nm) reached MFI values higher than the control.

Then we exposed the macrophages to both plastic beads and LPS. The same trends were observed as for cells exposed to plastic beads alone, but with stronger effects, so that highly phagocytic cells reached higher-than-control MFI values for all beads types except the largest ones.

As for other markers examined, this surface expression profile suggested variable physiological effects depending on the amount of plastic beads internalized, and also variable responses to LPS (and thus to bacterial infections) with variable amounts of internalized plastics.

## 5 Conclusions

When taken collectively, our results show that cells heavily loaded with plastic particles show a number of disturbances that may impact their function and beyond, the immune system homeostasis and the homeostasis of the surrounding tissue. For example, the mitochondrial dysfunction and the associated oxidative stress may impact the surrounding tissue by exposing it to higher-than-normal values of reactive oxygen species. The cells heavily loaded with plastics also express lower levels of the LFA-1 integrin and higher levels of PD-L1, suggesting an overall TAM-like phenotype and thus an altered function of the immune system.

These findings shift the key question on whether such heavily plastic-loaded macrophages can exist *in vivo*. This question arises from the fact that plastic contamination is clearly a low-dose, repeated contamination, so that accumulation in macrophages requires a number of conditions. The first condition is of course that plastics are not degraded in the macrophages, which we showed, as least for the polystyrene studied here.

There are however means to eliminate permanent particles, as shown for the respiratory exposure to some metallic particles (e.g. in (74, 75)). In this case, the elimination proceeds via the mucociliary escalator, by which particles-loaded macrophages are eliminated as a whole from the body, and replaced by new, particles-free macrophages. With the now known dynamics of alveolar macrophages (76), this elimination process may proceed either by asymmetrical mitosis (77, 78), leading to a heavily loaded daughter cell that is eliminated and a less loaded cell that is kept in place, or by eliminating the original macrophage as a whole and replacing it by a monocyte-derived one.

This process requires however that the particle is not too toxic to the macrophages and small enough to be eliminated. Such counter examples exist for example for asbestos, which fibers are too large to be eliminated with single macrophages, or for crystalline silica and beryllium, which are too toxic for macrophages (79–81), so that they die *in situ* and are replaced by new macrophages. During this process pro-inflammatory cytokines and reactive oxygen species are liberated (80–82), which created the tissue damaging situation leading to the pathology.

The favorable situation represented by the mucociliary escalator (or its equivalent) does not exist in all body locations where macrophages are present. The most spectacular counter example is represented by dermal macrophages and tattoos, where the short-lived, pigment-loaded dermal macrophages die in situ, liberating the pigment particles that are recaptured by fresh macrophages, leading to the permanence of the tattoo (27). Quite interestingly, dermal or intramuscular injection of persistent particles leads to detection in distal organs, and this has been observed for tattoos and for other particles (58, 83) It is not known yet whether this presence of particles in distal organs comes from:

-particles that have not been captured by the proximal macrophages upon their entry in the body, have been carried out in the lymph and have reached secondary lymphoid organs where they have been captured

-particles that have escaped one of this liberation-recapture cycles and have been recaptured distally

-migrating particles-loaded macrophages. Interestingly, it has been observed that plastic particles increase the chemotactic mobility of macrophages (22), and that macrophages entering the blood flow can relocate to several macrophages-rich organs (84).

The fact that plastic particles cross the intestinal barrier (10, 34, 85) can be found in distant organs upon oral administration (e.g. in (86, 87) argues in favor of a situation close to the one encountered for dermal macrophages. This correlates with the short lifespan of intestinal macrophages (88), comparable to the one of dermal macrophages (27), and opposite to the long lifespan of alveolar macrophages (89). It is thus likely that by a combination of regular intake and negligible elimination, an increasing population of highly plastic-loaded macrophages may appear upon time and regular ingestion of micro and nano plastics, with the potential deleterious consequences involved by the changes described in this study.

## Data availability statement

The raw data supporting the conclusions of this article will be made available by the authors, without undue reservation.

## Author contributions

VC-F carried out the cell culture, the flow cytometry experiments and the fluorophore leakage experiments. The flow cytometry experiments were analyzed by VC-F, BD, and TR. MV, AT, OT, and BD performed the beads characterization experiments. BD and AT performed the confocal microscopy experiments. BD suggested the core concept of subpopulations sorting. TR conducted and supervised the study, obtained the funding, wrote the initial version of the manuscript and handled the revision process. All authors contributed to the article and approved the submitted version.

## References

[1] Lehner R Weder C Petri-Fink A Rothen-Rutishauser B. Emergence of nanoplastic in the environment and possible impact on human health. Environ Sci Technol (2019) 53:1748–65. doi: 10.1021/acs.est.8b05512 30629421

[2] Fan YV Jiang P Tan RR Aviso KB You F Zhao X. Forecasting plastic waste generation and interventions for environmental hazard mitigation. J Hazard Mater (2022) 424:127330. doi: 10.1016/j.jhazmat.2021.127330 34600379

[3] Collard F Gasperi J Gilbert B Eppe G Azimi S Rocher V. Anthropogenic particles in the stomach contents and liver of the freshwater fish squalius cephalus. Sci Total Environ (2018) 643:1257–64. doi: 10.1016/j.scitotenv.2018.06.313 30189542

[4] Senathirajah K Attwood S Bhagwat G Carbery M Wilson S Palanisami T. Estimation of the mass of microplastics ingested - a pivotal first step towards human health risk assessment. J Hazard Mater (2021) 404:124004. doi: 10.1016/j.jhazmat.2020.124004 33130380

[5] Schwabl P Köppel S Königshofer P Bucsics T Trauner M Reiberger T. Detection of various microplastics in human stool: a prospective case series. Ann Internal Med (2019) 171:453. doi: 10.7326/M19-0618 31476765

[6] Amato-Lourenço LF Carvalho-Oliveira R Júnior GR Dos Santos Galvão L Ando RA Mauad T. Presence of airborne microplastics in human lung tissue. J Hazard Mater (2021) 416:126124. doi: 10.1016/j.jhazmat.2021.126124 34492918

[7] Horvatits T Tamminga M Liu B Sebode M Carambia A Fischer L. Microplastics detected in cirrhotic liver tissue. EBioMedicine (2022) 82:104147. doi: 10.1016/j.ebiom.2022.104147 35835713 PMC9386716

[8] Ragusa A Svelato A Santacroce C Catalano P Notarstefano V Carnevali O. Plasticenta: first evidence of microplastics in human placenta. Environ Int (2021) 146:106274. doi: 10.1016/j.envint.2020.106274 33395930

[9] Deng Y Zhang Y Lemos B Ren H. Tissue accumulation of microplastics in mice and biomarker responses suggest widespread health risks of exposure. Sci Rep (2017) 7:46687. doi: 10.1038/srep46687 28436478 PMC5402289

[10] Stock V Böhmert L Lisicki E Block R Cara-Carmona J Pack LK. Uptake and effects of orally ingested polystyrene microplastic particles *in vitro* and *in vivo* . Arch Toxicol (2019) 93:1817–33. doi: 10.1007/s00204-019-02478-7 31139862

[11] Domenech J Hernández A Rubio L Marcos R Cortés C. Interactions of polystyrene nanoplastics with *in vitro* models of the human intestinal barrier. Arch Toxicol (2020) 94:2997–3012. doi: 10.1007/s00204-020-02805-3 32592077

[12] Busch M Bredeck G Kämpfer AAM Schins RPF. Investigations of acute effects of polystyrene and polyvinyl chloride micro- and nanoplastics in an advanced *in vitro* triple culture model of the healthy and inflamed intestine. Environ Res (2021) 193:110536. doi: 10.1016/j.envres.2020.110536 33253701

[13] Leslie HA van Velzen MJM Brandsma SH Vethaak AD Garcia-Vallejo JJ Lamoree MH. Discovery and quantification of plastic particle pollution in human blood. Environ Int (2022) 163:107199. doi: 10.1016/j.envint.2022.107199 35367073

[14] Champion JA Walker A Mitragotri S. Role of particle size in phagocytosis of polymeric microspheres. Pharm Res (2008) 25:1815–21. doi: 10.1007/s11095-008-9562-y PMC2793372 18373181

[15] Chikaura H Nakashima Y Fujiwara Y Komohara Y Takeya M Nakanishi Y. Effect of particle size on biological response by human monocyte-derived macrophages. Biosurface Biotribology (2016) 2:18–25. doi: 10.1016/j.bsbt.2016.02.003

[16] Sadauskas E Wallin H Stoltenberg M Vogel U Doering P Larsen A. Kupffer cells are central in the removal of nanoparticles from the organism. Part Fibre Toxicol (2007) 4:10. doi: 10.1186/1743-8977-4-10 17949501 PMC2146996

[17] Sica A Mantovani A. Macrophage plasticity and polarization: *in vivo* veritas. J Clin Invest (2012) 122:787–95. doi: 10.1172/JCI59643 PMC3287223 22378047

[18] Jablonski KA Amici SA Webb LM Ruiz-Rosado J de D Popovich PG Partida-Sanchez S. Novel markers to delineate murine M1 and M2 macrophages. PloS One (2015) 10:e0145342. doi: 10.1371/journal.pone.0145342 26699615 PMC4689374

[19] Orecchioni M Ghosheh Y Pramod AB Ley K. Macrophage polarization: different gene signatures in M1(LPS+) vs. classically and M2(LPS–) vs. alternatively activated macrophages. Front Immunol (2019) 10:1084. doi: 10.3389/fimmu.2019.01084 31178859 PMC6543837

[20] Kubota K Moriyama M Furukawa S Rafiul HASM Maruse Y Jinno T. CD163+CD204+ tumor-associated macrophages contribute to T cell regulation via interleukin-10 and PD-L1 production in oral squamous cell carcinoma. Sci Rep (2017) 7:1755. doi: 10.1038/s41598-017-01661-z 28496107 PMC5431876

[21] Nagano M Saito K Kozuka Y Ichishi M Yuasa H Noro A. CD204-positive macrophages accumulate in breast cancer tumors with high levels of infiltrating lymphocytes and programmed death ligand-1 expression. Oncol Lett (2020) 21:1–1. doi: 10.3892/ol.2020.12297 33262828 PMC7693484

[22] Prietl B Meindl C Roblegg E Pieber TR Lanzer G Fröhlich E. Nano-sized and micro-sized polystyrene particles affect phagocyte function. Cell Biol Toxicol (2014) 30:1–16. doi: 10.1007/s10565-013-9265-y 24292270 PMC4434214

[23] Hu Q Wang H He C Jin Y Fu Z. Polystyrene nanoparticles trigger the activation of p38 MAPK and apoptosis via inducing oxidative stress in zebrafish and macrophage cells. Environ Pollut (2021) 269:116075. doi: 10.1016/j.envpol.2020.116075 33316494

[24] Fuchs A-K Syrovets T Haas KA Loos C Musyanovych A Mailänder V. Carboxyl- and amino-functionalized polystyrene nanoparticles differentially affect the polarization profile of M1 and M2 macrophage subsets. Biomaterials (2016) 85:78–87. doi: 10.1016/j.biomaterials.2016.01.064 26854393

[25] Collin-Faure V Dalzon B Devcic J Diemer H Cianférani S Rabilloud T. Does size matter? a proteomics-informed comparison of the effects of polystyrene beads of different sizes on macrophages. Environ Sci Nano (2022) 9:2827–40. doi: 10.1039/D2EN00214K

[26] Sadauskas E Danscher G Stoltenberg M Vogel U Larsen A Wallin H. Protracted elimination of gold nanoparticles from mouse liver. Nanomedicine (2009) 5:162–9. doi: 10.1016/j.nano.2008.11.002 19217434

[27] Baranska A Shawket A Jouve M Baratin M Malosse C Voluzan O. Unveiling skin macrophage dynamics explains both tattoo persistence and strenuous removal. J Exp Med (2018) 215:1115–33. doi: 10.1084/jem.20171608 PMC5881467 29511065

[28] Moon E-Y Yi G-H Kang J-S Lim J-S Kim H-M Pyo S. An increase in mouse tumor growth by an *in vivo* immunomodulating effect of titanium dioxide nanoparticles. J Immunotoxicol (2011) 8:56–67. doi: 10.3109/1547691X.2010.543995 21288165

[29] Huang C Sun M Yang Y Wang F Ma X Li J. Titanium dioxide nanoparticles prime a specific activation state of macrophages. Nanotoxicology (2017) 11:737–50. doi: 10.1080/17435390.2017.1349202 28669258

[30] Devcic J Dussol M Collin-Faure V Pérard J Fenel D Schoehn G. Immediate and sustained effects of cobalt and zinc-containing pigments on macrophages. Front Immunol (2022) 13:865239. doi: 10.3389/fimmu.2022.865239 35928812 PMC9343594

[31] Kedzierski M Palazot M Soccalingame L Falcou-Préfol M Gorsky G Galgani F. Chemical composition of microplastics floating on the surface of the Mediterranean Sea. Mar Pollut Bull (2022) 174:113284. doi: 10.1016/j.marpolbul.2021.113284 34995887

[32] Lambert S Wagner M. Characterisation of nanoplastics during the degradation of polystyrene. Chemosphere (2016) 145:265–8. doi: 10.1016/j.chemosphere.2015.11.078 PMC5250697 26688263

[33] Ter Halle A Jeanneau L Martignac M Jardé E Pedrono B Brach L. Nanoplastic in the north Atlantic subtropical gyre. Environ Sci Technol (2017) 51:13689–97. doi: 10.1021/acs.est.7b03667 29161030

[34] Hodges GM Carr EA Hazzard RA Carr KE. Uptake and translocation of microparticles in small intestine. morphology and quantification of particle distribution. Dig Dis Sci (1995) 40:967–75. doi: 10.1007/BF02064184 7729286

[35] Desai MP Labhasetwar V Amidon GL Levy RJ. Gastrointestinal uptake of biodegradable microparticles: effect of particle size. Pharm Res (1996) 13:1838–45. doi: 10.1023/a:1016085108889 8987081

[36] Khan J Iiboshi Y Nezu R Chen K Cui L Yoshida H. Total parenteral nutrition increases uptake of latex beads by peyer’s patches. JPEN J Parenter Enteral Nutr (1997) 21:31–5. doi: 10.1177/014860719702100131 9002082

[37] Torres A Dalzon B Collin-Faure V Rabilloud T. Repeated vs. acute exposure of RAW264.7 mouse macrophages to silica nanoparticles: a bioaccumulation and functional change study. Nanomaterials (2020) 10:215. doi: 10.3390/nano10020215 32012675 PMC7074975

[38] Dalzon B Torres A Devcic J Fenel D Sergent J-A Rabilloud T. A low-serum culture system for prolonged in vitro toxicology experiments on a macrophage system. Front Toxicol (2021) 3:780778. doi: 10.3389/ftox.2021.780778 35295137 PMC8915817

[39] Keij JF Bell-Prince C Steinkamp JA. Staining of mitochondrial membranes with 10-nonyl acridine orange, MitoFluor green, and MitoTracker green is affected by mitochondrial membrane potential altering drugs. Cytometry (2000) 39:203–10. doi: 10.1002/(sici)1097-0320(20000301)39:3<203::aid-cyto5>3.0.co;2-z 10685077

[40] Han MG Foulger SH. Crystalline colloidal arrays composed of Poly(3,4-ethylenedioxythiophene)-Coated polystyrene particles with a stop band in the visible regime. Adv Mater (2004) 16:231–4. doi: 10.1002/adma.200305642

[41] Torres A Collin-Faure V Diemer H Moriscot C Fenel D Gallet B. Repeated exposure of macrophages to synthetic amorphous silica induces adaptive proteome changes and a moderate cell activation. Nanomaterials (Basel) (2022) 12:1424. doi: 10.3390/nano12091424 35564134 PMC9105884

[42] Yamazaki T Akiba H Iwai H Matsuda H Aoki M Tanno Y. Expression of programmed death 1 ligands by murine T cells and APC. J Immunol (2002) 169:5538–45. doi: 10.4049/jimmunol.169.10.5538 12421930

[43] Sun C Mezzadra R Schumacher TN. Regulation and function of the PD-L1 checkpoint. Immunity (2018) 48:434–52. doi: 10.1016/j.immuni.2018.03.014 PMC7116507 29562194

[44] Strom KA Garg BD Johnson JT D’Arcy JB Smiler KL. Inhaled particle retention in rats receiving low exposures of diesel exhaust. J Toxicol Environ Health (1990) 29:377–98. doi: 10.1080/15287399009531399 1691304

[45] Witkiewicz H Vidovszky T Turner RT Rock MG Morrey BF Bolander ME. Fate of ultrahigh molecular weight polyethylene (UHMW-PE) wear debris in patients with hip implants. Tech Orthop (1976) (1993) 8:254–61. doi: 10.1097/00013611-199300840-00006 11539273

[46] Gilberti RM Joshi GN Knecht DA. The phagocytosis of crystalline silica particles by macrophages. Am J Respir Cell Mol Biol (2008) 39:619–27. doi: 10.1165/rcmb.2008-0046OC PMC2574531 18556590

[47] Tran CL Jones AD Donaldson K. Evidence of overload, dissolution and breakage of MMVF10 fibres in the RCC chronic inhalation study. Exp Toxicol Pathol (1996) 48:500–4. doi: 10.1016/S0940-2993(96)80066-2 8954332

[48] Warheit DB Hansen JF Yuen IS Kelly DP Snajdr SI Hartsky MA. Inhalation of high concentrations of low toxicity dusts in rats results in impaired pulmonary clearance mechanisms and persistent inflammation. Toxicol Appl Pharmacol (1997) 145:10–22. doi: 10.1006/taap.1997.8102 9221819

[49] Fujimura N. Pathology and pathophysiology of pneumoconiosis. Curr Opin Pulm Med (2000) 6:140–4. doi: 10.1097/00063198-200003000-00010 10741774

[50] Nakano-Narusawa Y Yokohira M Yamakawa K Saoo K Imaida K Matsuda Y. Single intratracheal quartz instillation induced chronic inflammation and tumourigenesis in rat lungs. Sci Rep (2020) 10:6647. doi: 10.1038/s41598-020-63667-4 32313071 PMC7170867

[51] Norboo T Angchuk PT Yahya M Kamat SR Pooley FD Corrin B. Silicosis in a Himalayan village population: role of environmental dust. Thorax (1991) 46:341–3. doi: 10.1136/thx.46.5.341 PMC463131 2068689

[52] Korshunov SS Skulachev VP Starkov AA. High protonic potential actuates a mechanism of production of reactive oxygen species in mitochondria. FEBS Lett (1997) 416:15–8. doi: 10.1016/s0014-5793(97)01159-9 9369223

[53] Starkov AA Fiskum G. Regulation of brain mitochondrial H2O2 production by membrane potential and NAD(P)H redox state. J Neurochem (2003) 86:1101–7. doi: 10.1046/j.1471-4159.2003.01908.x 12911618

[54] Hu Z Yu F Gong P Qiu Y Zhou W Cui Y. Subneurotoxic copper(II)-induced NF-κB-dependent microglial activation is associated with mitochondrial ROS. Toxicol Appl Pharmacol (2014) 276:95–103. doi: 10.1016/j.taap.2014.01.020 24530511

[55] Pomothy J Szombath G Rokonál P Mátis G Neogrády Z Steinmetzer T. The impact of acute matriptase inhibition in hepatic inflammatory models. BioMed Res Int (2016) 2016:6306984. doi: 10.1155/2016/6306984 27642598 PMC5013213

[56] Castranova V. Generation of oxygen radicals and mechanisms of injury prevention. Environ Health Perspect (1994) 102 Suppl 10:65–8. doi: 10.1289/ehp.94102s1065 PMC1566974 7705309

[57] Liang B Zhong Y Huang Y Lin X Liu J Lin L. Underestimated health risks: polystyrene micro- and nanoplastics jointly induce intestinal barrier dysfunction by ROS-mediated epithelial cell apoptosis. Part Fibre Toxicol (2021) 18:20. doi: 10.1186/s12989-021-00414-1 34098985 PMC8186235

[58] Schreiver I Hesse B Seim C Castillo-Michel H Villanova J Laux P. Synchrotron-based ν-XRF mapping and μ-FTIR microscopy enable to look into the fate and effects of tattoo pigments in human skin. Sci Rep (2017) 7:11395. doi: 10.1038/s41598-017-11721-z 28900193 PMC5595966

[59] Vandendriessche S Cambier S Proost P Marques PE. Complement receptors and their role in leukocyte recruitment and phagocytosis. Front Cell Dev Biol (2021) 9:624025. doi: 10.3389/fcell.2021.624025 33644062 PMC7905230

[60] Ehirchiou D Xiong Y Xu G Chen W Shi Y Zhang L. CD11b facilitates the development of peripheral tolerance by suppressing Th17 differentiation. J Exp Med (2007) 204:1519–24. doi: 10.1084/jem.20062292 PMC2118631 17562817

[61] Han C Jin J Xu S Liu H Li N Cao X. Integrin CD11b negatively regulates TLR-triggered inflammatory responses by activating syk and promoting degradation of MyD88 and TRIF via cbl-b. Nat Immunol (2010) 11:734–42. doi: 10.1038/ni.1908 20639876

[62] Bednarczyk M Stege H Grabbe S Bros M. β2 integrins-Multi-Functional leukocyte receptors in health and disease. Int J Mol Sci (2020) 21:E1402. doi: 10.3390/ijms21041402 PMC7073085 32092981

[63] Walling BL Kim M. LFA-1 in T cell migration and differentiation. Front Immunol (2018) 9:952. doi: 10.3389/fimmu.2018.00952 29774029 PMC5943560

[64] Winkler HC Kornprobst J Wick P von Moos LM Trantakis I Schraner EM. MyD88-dependent pro-interleukin-1β induction in dendritic cells exposed to food-grade synthetic amorphous silica. Part Fibre Toxicol (2017) 14:21. doi: 10.1186/s12989-017-0202-8 28645296 PMC5481969

[65] Feray A Guillet E Szely N Hullo M Legrand F-X Brun E. Synthetic amorphous silica nanoparticles promote human dendritic cell maturation and CD4 + T-lymphocyte activation. Toxicol Sci (2021) 185:105–16. doi: 10.1093/toxsci/kfab120 34633463

[66] Kelley JL Ozment TR Li C Schweitzer JB Williams DL. Scavenger receptor-a (CD204): a two-edged sword in health and disease. Crit Rev Immunol (2014) 34:241–61. doi: 10.1615/CritRevImmunol.2014010267 PMC4191651 24941076

[67] Lunov O Syrovets T Loos C Beil J Delacher M Tron K. Differential uptake of functionalized polystyrene nanoparticles by human macrophages and a monocytic cell line. ACS Nano (2011) 5:1657–69. doi: 10.1021/nn2000756 21344890

[68] Florance I Ramasubbu S Mukherjee A Chandrasekaran N. Polystyrene nanoplastics dysregulate lipid metabolism in murine macrophages *in vitro* . Toxicology (2021) 458:152850. doi: 10.1016/j.tox.2021.152850 34217793

[69] Frick SU Bacher N Baier G Mailänder V Landfester K Steinbrink K. Functionalized polystyrene nanoparticles trigger human dendritic cell maturation resulting in enhanced CD4+ T cell activation. Macromol Biosci (2012) 12:1637–47. doi: 10.1002/mabi.201200223 23042770

[70] Nishiya T Kajita E Miwa S Defranco AL. TLR3 and TLR7 are targeted to the same intracellular compartments by distinct regulatory elements. J Biol Chem (2005) 280:37107–17. doi: 10.1074/jbc.M504951200 16105838

[71] Kim S-J Chen Z Essani AB Elshabrawy HA Volin MV Volkov S. Identification of a novel toll-like receptor 7 endogenous ligand in rheumatoid arthritis synovial fluid that can provoke arthritic joint inflammation. Arthritis Rheumatol (2016) 68:1099–110. doi: 10.1002/art.39544 PMC5221504 26662519

[72] Van Raemdonck K Umar S Palasiewicz K Romay B Volkov S Arami S. TLR7 endogenous ligands remodel glycolytic macrophages and trigger skin-to-joint crosstalk in psoriatic arthritis. Eur J Immunol (2021) 51:714–20. doi: 10.1002/eji.202048690 PMC10018531 33079387

[73] Rawle DJ Dumenil T Tang B Bishop CR Yan K Le TT. Microplastic consumption induces inflammatory signatures in the colon and prolongs a viral arthritis. Sci Total Environ (2022) 809:152212. doi: 10.1016/j.scitotenv.2021.152212 34890673

[74] Semmler-Behnke M Takenaka S Fertsch S Wenk A Seitz J Mayer P. Efficient elimination of inhaled nanoparticles from the alveolar region: evidence for interstitial uptake and subsequent reentrainment onto airways epithelium. Environ Health Perspect (2007) 115:728–33. doi: 10.1289/ehp.9685 PMC1867986 17520060

[75] Kreyling WG Holzwarth U Schleh C Hirn S Wenk A Schäffler M. Quantitative biokinetics over a 28 day period of freshly generated, pristine, 20 nm titanium dioxide nanoparticle aerosols in healthy adult rats after a single two-hour inhalation exposure. Part Fibre Toxicol (2019) 16:29. doi: 10.1186/s12989-019-0303-7 31288843 PMC6617842

[76] Gordon S Plüddemann A. Tissue macrophages: heterogeneity and functions. BMC Biol (2017) 15:53. doi: 10.1186/s12915-017-0392-4 28662662 PMC5492929

[77] Errington RJ Brown MR Silvestre O Njoh KL Chappell SC Khan I. Single cell nanoparticle tracking to model cell cycle dynamics and compartmental inheritance. Cell Cycle (2010) 9:121–30. doi: 10.4161/cc.9.1.10246 20016285

[78] Ye D Li M Feng K Zhang Y Sheng J Gu N. Long-term fate tracking and quantitative analyzing of nanoparticles in stem cells with bright-field microscopy. Nano Today (2022) 44:101506. doi: 10.1016/j.nantod.2022.101506

[79] Larivée P Cantin A Dufresne A Bégin R. Enzyme activities of lung lavage in silicosis. Lung (1990) 168:151–8. doi: 10.1007/BF02719686 2114508

[80] Sawyer RT Dobis DR Goldstein M Velsor L Maier LA Fontenot AP. Beryllium-stimulated reactive oxygen species and macrophage apoptosis. Free Radic Biol Med (2005) 38:928–37. doi: 10.1016/j.freeradbiomed.2004.12.014 15749389

[81] Nie W Lan T Yuan X Luo M Shen G Yu J. Crystalline silica induces macrophage necrosis and causes subsequent acute pulmonary neutrophilic inflammation. Cell Biol Toxicol (2022) 38:591–609. doi: 10.1007/s10565-021-09620-1 34170461

[82] Sawyer RT Kittle LA Hamada H Newman LS Campbell PA. Beryllium-stimulated production of tumor necrosis factor-alpha by a mouse hybrid macrophage cell line. Toxicology (2000) 143:235–47. doi: 10.1016/s0300-483x(99)00182-1 10755710

[83] Khan Z Combadière C Authier F-J Itier V Lux F Exley C. Slow CCL2-dependent translocation of biopersistent particles from muscle to brain. BMC Med (2013) 11:99. doi: 10.1186/1741-7015-11-99 23557144 PMC3616851

[84] Dalzon B Guidetti M Testemale D Reymond S Proux O Vollaire J. Utility of macrophages in an antitumor strategy based on the vectorization of iron oxide nanoparticles. Nanoscale (2019) 11:9341–52. doi: 10.1039/c8nr03364a 30950461

[85] Jani P Halbert GW Langridge J Florence AT. Nanoparticle uptake by the rat gastrointestinal mucosa: quantitation and particle size dependency. J Pharm Pharmacol (1990) 42:821–6. doi: 10.1111/j.2042-7158.1990.tb07033.x 1983142

[86] Walczak AP Hendriksen PJM Woutersen RA van der Zande M Undas AK Helsdingen R. Bioavailability and biodistribution of differently charged polystyrene nanoparticles upon oral exposure in rats. J Nanopart Res (2015) 17:231. doi: 10.1007/s11051-015-3029-y 26028989 PMC4440892

[87] Schwarzfischer M Niechcial A Lee SS Sinnet B Wawrzyniak M Laimbacher A. Ingested nano- and microsized polystyrene particles surpass the intestinal barrier and accumulate in the body. NanoImpact (2022) 25:100374. doi: 10.1016/j.impact.2021.100374 35559880

[88] Bain CC Bravo-Blas A Scott CL Perdiguero EG Geissmann F Henri S. Constant replenishment from circulating monocytes maintains the macrophage pool in the intestine of adult mice. Nat Immunol (2014) 15:929–37. doi: 10.1038/ni.2967 PMC4169290 25151491

[89] Murphy J Summer R Wilson AA Kotton DN Fine A. The prolonged life-span of alveolar macrophages. Am J Respir Cell Mol Biol (2008) 38:380–5. doi: 10.1165/rcmb.2007-0224RC PMC2274942 18192503

