# Supplementary material for: The internal dose makes the poison: higher internalization of polystyrene particles induce increased perturbation of macrophages
Source: Front Immunol. 2023 May 12;14:1092743. doi: 10.3389/fimmu.2023.1092743 (PMC10213243; doi:10.3389/fimmu.2023.1092743)
Supplement: Supplementary file 1 [file Image_1.pdf]

Supplementary Figure 1

Methods

Infrared measurements :

Infrared (IR) spectra were recorded with a PerkinElmer Spectrum 100 FT-IR spectrometer equipped with a Pike MIRacle attenuated total reflectance (ATR) module, in the 4000-600 cm^{-1} range. For the measurements a Ge crystal was mounted on the ATR module.

Sample preparation:

500µl of polystyrene beads dispersion (1 or 2.5 mg/ml depending on the beads) were diluted in 1 ml EtOH and centrifuged at 15000g for 1 hour to pellet the beads. Two aliquots were needed for the 40-90 nm beads, which are sold as a 1 mg/ml dispersion. After centrifugation, all but 300 µl of the supernatant was removed and the beads dispersed in the remaining EtOH. 2.5 µL of the resulting suspension was drop-casted on the Ge crystal. The spectra were recorded after complete evaporation of the solvents, under mild pressure (built-in ATR press).

Results

The spectra, displayed below, show the characteristic peaks of polystyrene, as described in the literature (40).

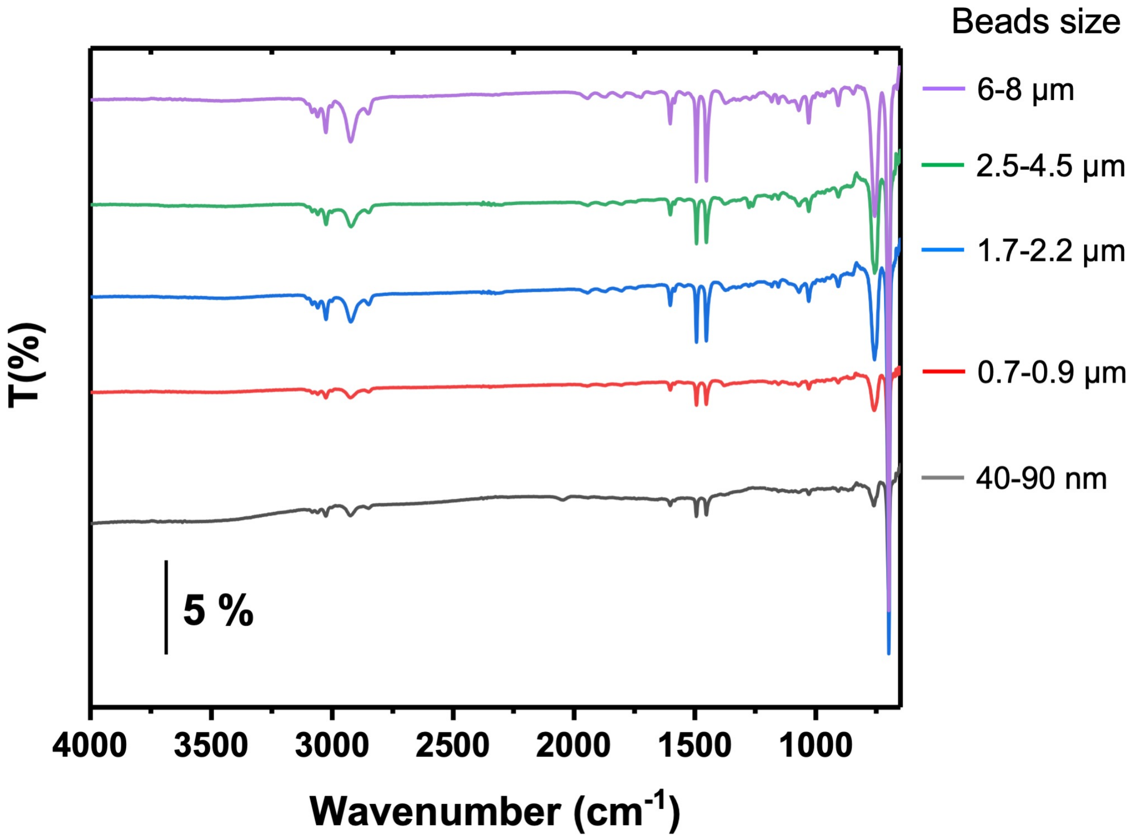

Supplementary Figure 1: FTIR spectra of the polystyrene beads used in the study
