# Supplementary material for: The internal dose makes the poison: higher internalization of polystyrene particles induce increased perturbation of macrophages
Source: Front Immunol. 2023 May 12;14:1092743. doi: 10.3389/fimmu.2023.1092743 (PMC10213243; doi:10.3389/fimmu.2023.1092743)
Supplement: Supplementary file 2 [file Image_2.pdf]

Supplementary Figure 2

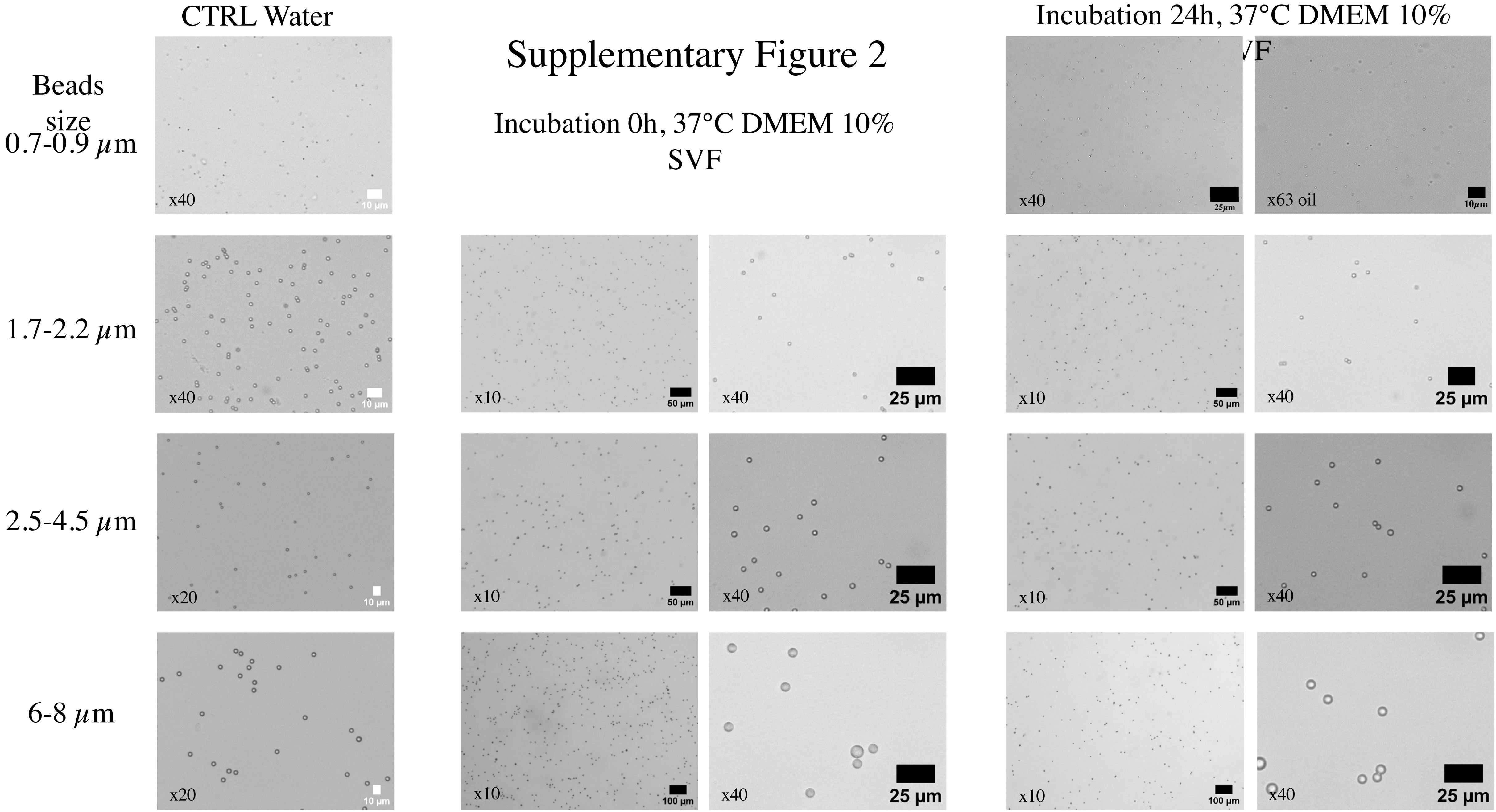

Supplementary Figure 2: optical microscopy images of the beads used in the study
