# Supplementary material for: The internal dose makes the poison: higher internalization of polystyrene particles induce increased perturbation of macrophages
Source: Front Immunol. 2023 May 12;14:1092743. doi: 10.3389/fimmu.2023.1092743 (PMC10213243; doi:10.3389/fimmu.2023.1092743)
Supplement: Supplementary file 3 [file Image_3.pdf]

Supplementary Figure 3

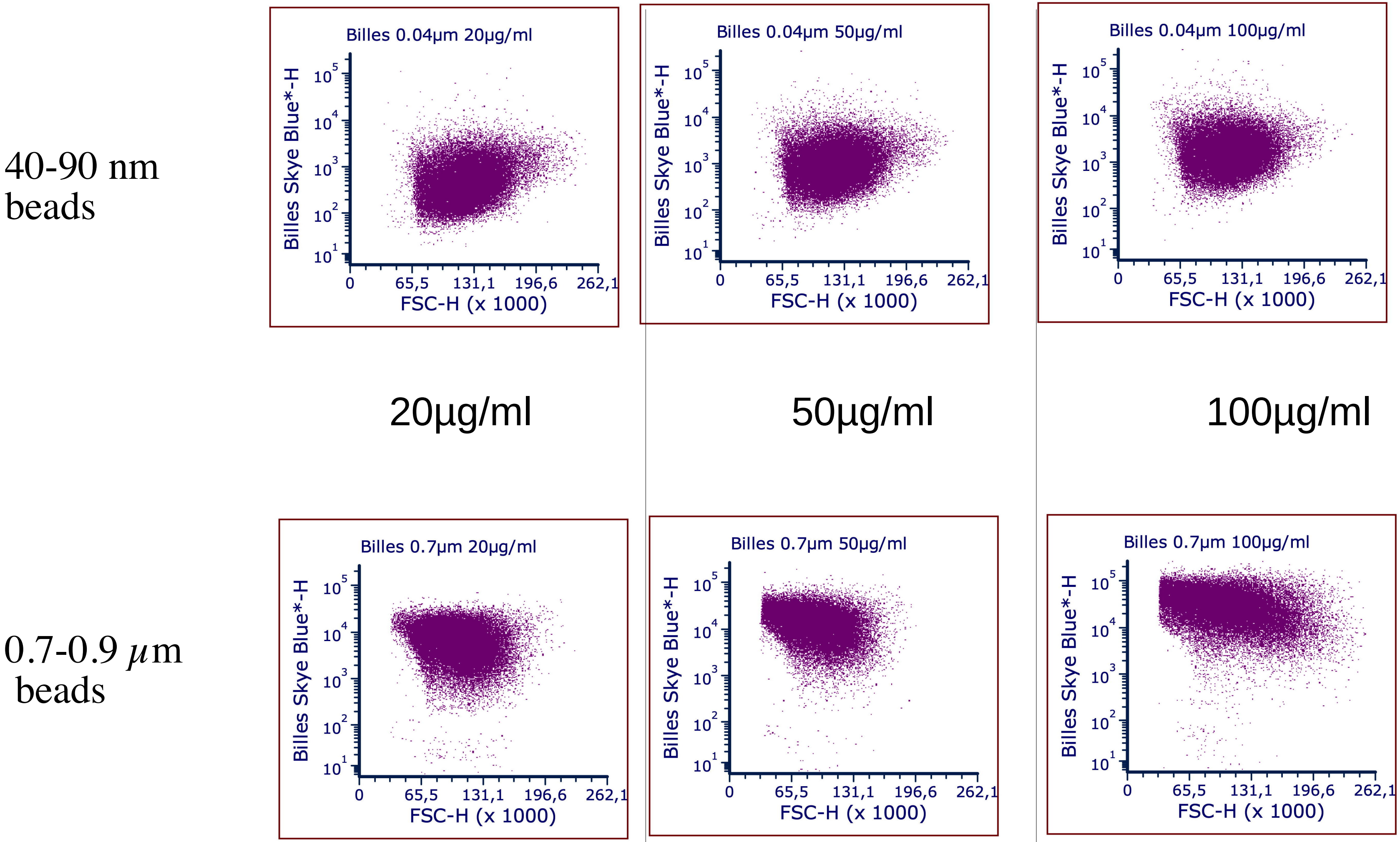

Supplementary Figure 3: flow cytometry diagrams of cells treated with various concentrations of beads
