# Supplementary material for: The internal dose makes the poison: higher internalization of polystyrene particles induce increased perturbation of macrophages
Source: Front Immunol. 2023 May 12;14:1092743. doi: 10.3389/fimmu.2023.1092743 (PMC10213243; doi:10.3389/fimmu.2023.1092743)
Supplement: Supplementary file 4 [file Image_4.pdf]

Supplementary Figure 4

Methods

Cells were treated with the various plastic beads as described in the material and methods. After 24 house of treatment, cells were sorted by flow cytometry using a Facs Melody (BD) instrument. Cell sorting is done in 3 or 4 groups according to the fluorescence intensity: negative cell population (when available), low intensity population, medium intensity and high intensity. After sorting and recovery of the cells (100000 cells in 1 mL), the cells suspensions were deposited on a sterile glass coverslip and incubated at 37°C for 3 to 4 hours to let the cells adhere to the slide. The cells are then rinsed in PBS twice and fixed with 4% PFA for 30 minutes at room temperature. The fixation is then maintained overnight at 4°C.
The next day, , after two washes, the cells were permeabilized in 0.1% Triton X100 for 5 minutes at room temperature. Two washes were again performed and cells were incubated with phalloidin-Atto 488 (catalogue number: 49409-10NMOL, Merck) at 250nM final concentration for 45 min at room temperature in the dark. Cells were washed again and nuclei were stained for 5 min with DAPI (1µg/mL final concentration). After two rinses with PBS, the glass coverslips were inverted, placed on microscope slides (Thermo Scientific, Waltham, MS, USA) using Vectashield mounting medium (Catalogue number: H-1000, Eurobio) and imaged using a Zeiss LSM 800 confocal microscope (Zeiss). Laser (source) of excitation used are the following : 405 nm for DAPI (blue emission), 488 nm for Atto 488 (green emission) and 633 nm for fluorescent Dark-red plastics beads (far red emission). Images obtained were processed using the FIJI software. The image settings are identical between the different negative, low, medium and high cell populations intensity selected.

Results

internalization-negative population | low-internalization population | medium-internalization population | high-internalization population

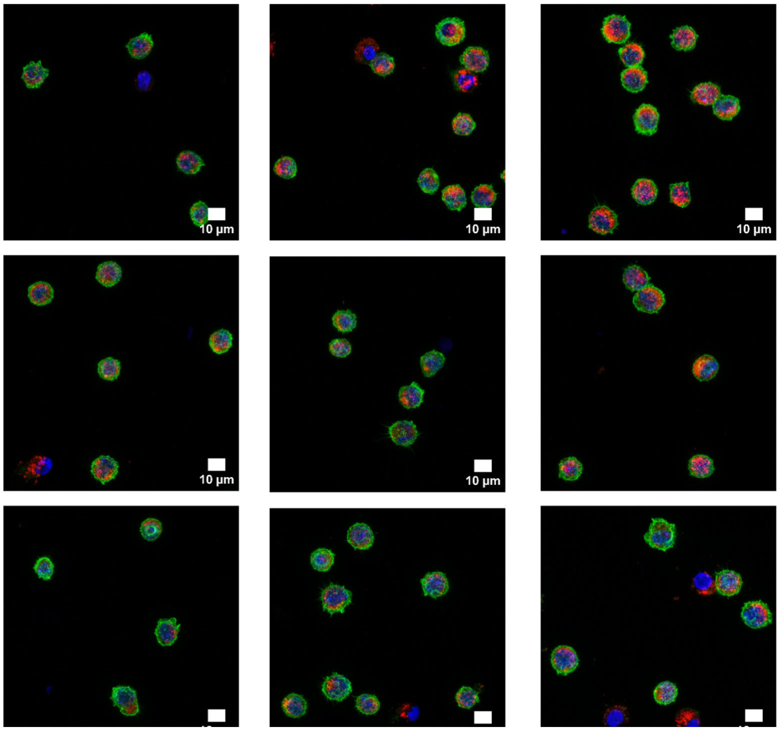

40-90 nm beads

Supplementary Figure 4A: confocal microscopy images of cells treated with 40-90 nm beads and sorted into subpopulations according to the fluorescence of the internalized beads

internalization-negative population | low-internalization population | medium-internalization population | high-internalization population

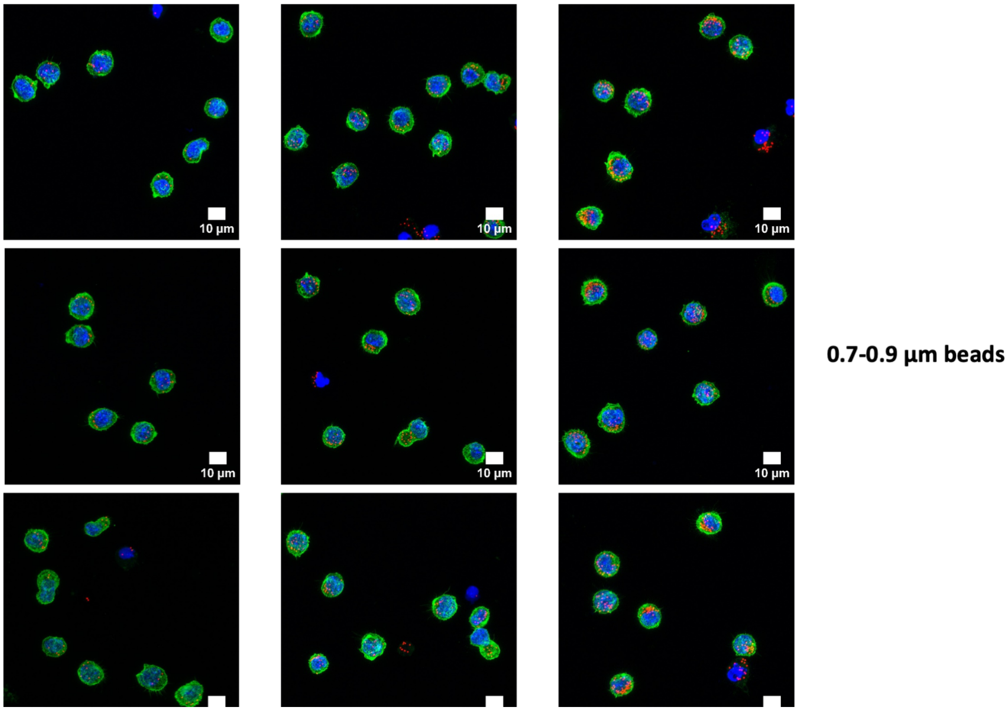

Supplementary Figure 4B: confocal microscopy images of cells treated with 40.7-0.9 µm beads and sorted into subpopulations according to the fluorescence of the internalized beads

internalization-negative population | low-internalization population | medium-internalization population | high-internalization population

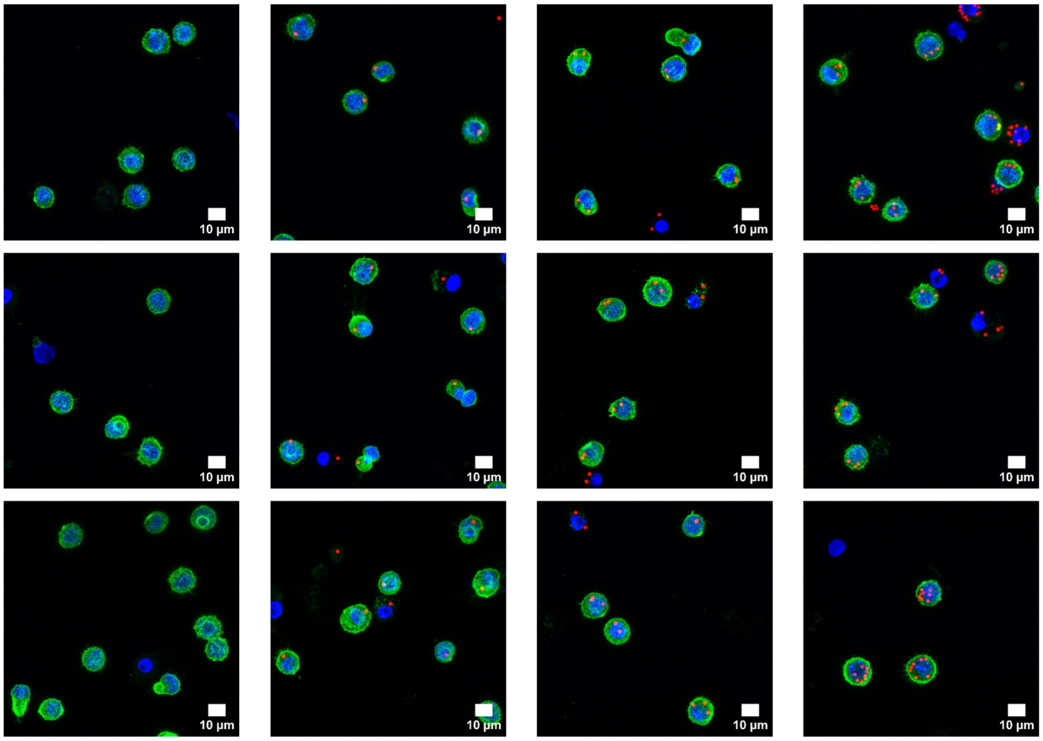

Supplementary Figure 4C: confocal microscopy images of cells treated with 1.7-2.2 µm beads and sorted into subpopulations according to the fluorescence of the internalized beads

Conclusions

Confocal microscopy confirms that the different subpopulations detected in flow cytometry do correspond to variable numbers of internalized beads
