# Supplementary material for: The internal dose makes the poison: higher internalization of polystyrene particles induce increased perturbation of macrophages
Source: Front Immunol. 2023 May 12;14:1092743. doi: 10.3389/fimmu.2023.1092743 (PMC10213243; doi:10.3389/fimmu.2023.1092743)
Supplement: Supplementary file 5 [file Table_1.pdf]

Supplementary table 1

Methods

Beads were suspended at the indicated concentrations in complete culture medium (DMEM+10% FBS) and incubated at 37°C for 24 hours.
At the end of the incubation period, the suspension was re-homogeneized by vortexing, and its fluorescence (Ex: 635nm, Em 665-740 nm) was measured on a DeNovix fluorometer.
The suspension was then centrifuged at 15,000g for 1 hour to collect the beads, and the fluorescence of the supernatant was measured using the same parameters as for the suspension. The ratio between the two fluorescence intensities gives the leakage of the beads

	Measure incubated suspension (24hrs) (corrected for DMEM background fluorescence)				
	DMEM CTL	Beads 0.7-0.9 µm 20µg/ml	beads 1.7-2.2 µm 100µg/ml	Beads 2.5-4.5 µm 20µg/ml	beads 6-8 µm 100µg/ml
	0	19054.4	6200	7968.4	133.5
	0	18792	5736.1	8025.1	114.3
	0	18972	5948.8	8016	114
	0	18920.5	5920.2	8264.2	116.4
mean	0	18934.7	5951.3	8068.4	119.6
std deviation	0	109.98	190.73	132.86	9.36

Measure post centrifugation (1h00 15,000 g) (corrected for DMEM background fluorescence)				
DMEM CTL	Beads 0.7-0.9 µm 20µg/ml	Beads 1.7-2.2 µm 100µg/ml	Beads 2.5-4.5 µm 20µg/ml	beads 6-8 µm 100µg/ml
0	89.7	68.6	12.2	-2.5
0	80.8	48.8	1.8	0.8
0	103.8	40.6	3.4	-2.3
0	165.6	49	17.2	7.8
0.0	110.0	51.8	8.7	0.95
0.00	38.27	11.90	7.31	4.81

	Leakage in percent (after 24 hours)			
	Beads 0.7-0.9 µm 20µg/ml	beads 1.7-2.2 µm 100µg/ml	Beads 2.5-4.5 µm 20µg/ml	beads 6-8 µm 100µg/ml
	0.47	1.11	0.15	-1.87
	0.43	0.85	0.02	0.70
	0.55	0.68	0.04	-2.02
	0.88	0.83	0.21	6.70
mean	0.58	0.87	0.11	0.88
std deviation	0.20	0.18	0.09	4.08
